# Global, regional and national burden of neck pain in children and adolescents: GBD 2021 systematic analysis

**DOI:** 10.3389/fneur.2025.1625954

**Published:** 2025-11-24

**Authors:** Chao Sun, Hao Lin, Zhiqiang Zhang, Xinyue Yang, Jiaming Zhou, Rui Wang, Yuan Xue

**Affiliations:** 1 Department of Orthopedics Surgery, Tianjin Medical University General Hospital, Tianjin, China; 2 Tianjin Key Laboratory of Spine and Spinal Cord, Tianjin Medical University General Hospital, Tianjin, China; 3 Tianjin Medical University, Tianjin, China; 4 Department of Orthopedic Surgery, Affiliated Hospital of Hebei University, Baoding, China

**Keywords:** neck pain, children, adolescents, global burden of disease, musculoskeletal disorders

## Abstract

**Background:**

Neck pain (NP) has emerged as a significant health concern among children and adolescents, with potential lifelong consequences. This study aimed to comprehensively assess the global, regional, and national burden of NP among this population using data from the 2021 study.

**Methods:**

Publicly available modeled data and methods from the Global Burden of Disease (GBD) study 2021 were used to systematically analyze and evaluate the global burden of NP among children and adolescents. Primary outcomes were NP related age-standardized prevalence, incidence, years lived with disability (YLDs), and average annual percentage change (AAPC).

**Results:**

Between 1990 and 2021, the global prevalence of NP among children and adolescents increased from 8.49 million to 10.32 million cases, though age-standardized rates remained relatively stable (AAPC 0.01%). The global incidence of NP among children and adolescents increased from 3.17 million cases in 1990 to 3.82 million in 2021, while the age-standardized incidence rate showed a minimal decrease (AAPC −0.02%). The burden of NP in terms of YLDs increased from 0.89 million in 1990 to 1.08 million in 2021, although the age-standardized YLD rate remained stable (AAPC 0%). A pronounced age gradient was observed, with rates in 2021 peaking in the 15–19 years group (1,012.03 per 100,000) compared to the 5–9 years group (97.44 per 100,000), though the youngest group showed the largest relative increases over time (AAPC 0.10%). Females consistently exhibited 33.1% higher prevalence than males. High sociodemographic index (SDI) regions demonstrated the highest prevalence rates, with Western Europe (984.25 per 100,000), High-income North America (827.69), and Central Europe (751.30) leading, though low SDI regions showed more rapid increases. At the national level, Italy, Norway, and Denmark had the highest rates, while Middle Eastern countries experienced the most rapid increases.

**Conclusion:**

Despite stable global age-standardized rates, the increasing absolute burden of NP among children and adolescents remains concerning. This study provides strong evidence that there are diverse changing patterns of NP burden across different sex, age, and SDI groups within children and adolescents.

## Introduction

Neck pain (NP) has emerged as a significant global health concern among children and adolescents, with evidence suggesting its origins often lie in these formative years (1–3). Beyond physical discomfort, pediatric NP negatively impacts academic performance, physical activity participation, and quality of life (4, 5). Psychologically, it associates with anxiety, depression, and sleep disturbances in a bidirectional relationship (6, 7). Socially, affected youth often withdraw from peer interactions (8), while economically, it creates substantial burdens through healthcare costs and parental work absenteeism (9, 10). Most concerning is the longitudinal trajectory — adolescents with NP face significantly higher risks of developing chronic pain syndromes as adults, affecting workforce participation and lifetime earning potential (11, 12).

Despite digital technology use, sedentary lifestyles, poor school ergonomics, and psychological stressors driving this trend (13, 14), the true global burden of pediatric NP remains inadequately quantified (15). In this study, we comprehensively assess NP burden in young populations worldwide by analyzing prevalence, incidence, and disability metrics across age, sex, sociodemographic index (SDI). These findings may inform healthcare policies, prevention programs, and age-appropriate clinical guidelines, addressing this emerging public health challenge before it translates into lifelong disability (16, 17).

## Methods

This study adheres to the Guidelines for Accurate and Transparent Health Estimates Reporting (GATHER) for reporting standards.

### Overview of GBD

The GBD 2021 study, conducted by the Institute for Health Metrics and Evaluation (IHME), represents the latest iteration of a comprehensive, data-driven initiative to assess the health status of populations worldwide. GBD 2021 analyzes 371 diseases and injuries, spanning 204 countries and territories and 811 subnational locations, to provide a detailed understanding of global, regional, and national health trends (18).

### Case definition and data sources

Per the GBD study’s criteria, NP was defined as pain in the cervical spine region, lasting for at least 24 h, with or without referred pain to the arms (18, 19). The data were available at the GBD 2021 websites^1^^,^^2^ (18, 20). GBD 2021 complied with the Guidelines for Accurate and Transparent Health Estimate Reporting statement (18).

### Data processing and disease model

Prevalence estimates were stratified by sex and age wherever possible. For studies reporting data with broad age ranges by sex or specific age ranges combining both sexes, sex-specific prevalence was derived using within-study sex ratios and uncertainty intervals. If these ratios were not available, sex-specific estimates were obtained through a meta-analysis using the Meta Regression-Bayesian, Regularized Trimmed (MR-BRT) approach. The resulting female-to-male ratio was 1.18. After applying bias adjustments, data for age groups spanning 15 years were divided into specific age categories (5–9, 10–14, and 15–19 years) based on prevalence age patterns estimated by the Bayesian meta-regression tool, DisMod-MR 2.1, designed for global burden of disease studies (18). Three additional covariates were incorporated for claims data from the United States (year 2000 and from 2010 onward) and Taiwan (province of China). However, due to the inability to establish reliable MR-BRT network crosswalk matches for Taiwan, its claims data were excluded from the final model. After adjusting for case definitions, outlier data were systematically removed. Excess mortality was set to zero in the DisMod model, and it was assumed there were no incident or prevalent NP cases before the age of five. A summary exposure value (SEV) scalar was applied as a country-specific covariate for NP, combining exposure measures for risks significantly affecting NP in the GBD framework, such as elevated body mass index (BMI) and occupational ergonomic exposure. The SEV boundary values were set between 0.75 and 1.25. Further details on data processing and the disease model can be found in reference (18). The data details, methodology for data quality and comparability, and statistical modeling for GBD 2021 have been previously explained (18).

### Years lived with disability

As no evidence for mortality from NP was found in the GBD Study, YLDs and disability-adjusted life years (DALYs) values were identical, and the term YLDs was used in this study. YLDs quantify non-fatal health loss from a disease or injury, calculated by multiplying the prevalence of NP among children and adolescents by its disability weight.

### Data extraction

We analyzed data from the 2021 GBD study, which was obtained from the Global Health Data Exchange. The data consisted of repeated cross-sectional datasets covering 204 countries and territories within 21 regions from 1990 to 2021, and included 371 diseases and injuries as well as 88 risk factors globally, including NP. From the GBD 2021 study, we extracted data on the prevalence, incidence and YLDs of NP among children and adolescents, stratified by countries and territories, regions, sex, and age. YLD quantifies non-fatal health loss from a disease or injury, calculated by multiplying the prevalence and incidence of NP among children and adolescents by its disability weight. The methodology employed in the Global Burden of Disease Study 2021 is described elsewhere (also see Supplementary file, methods section) (18).

### Study population

In this study, we selected populations with NP aged 5–19 years as our research subjects.

### Statistics

To describe the global burden of NP among children and adolescents, a descriptive study was conducted. We conducted a comparative analysis of the age-standardized prevalence, age-standardized incidence, and age standardized YLDs of NP in different age groups, sexes, regions, and countries. We estimated average annual percentage changes (AAPCs) by joinpoint regression to measure the temporal trend, and we further calculated the age standardized rates and corresponding 95% confidence intervals (CIs) based on the world standard population reported in the Global Burden of Disease Study 2021 for comparison between regions (18). These calculations were based on data on NP among children and adolescents obtained from the Global Burden of Disease Study. Our estimates per 100,000 persons are displayed using the equation, which has been described in detail in previous article (18).

AAPCs are the annual change percentages (increase, decrease, or no change), which are used to represent the average increase or rate of change of a specific variable over a specified period, transformed from the weighted average of the slope coefficients of the underlying join point regression model from 1990 to 2021 (18, 21). We considered the corresponding rate as being in an upward (or downward) trend if the annual percentage change estimates and 95% confidence intervals (CIs) were both >0 (or both <0).

All statistical analyses were conducted using R (version 4.2.3), Joinpoint Regression Software (version 5.0.2), and GraphPad Prism (version 8.0).

## Results

### Global trends

Between 1990 and 2021, the global prevalence of NP among children and adolescents increased from 8.49 million to 10.32 million. However, the age-standardized prevalence rate remained relatively stable, changing only slightly from 512.15 per 100,000 in 1990 to 513.37 per 100,000 in 2021, with an AAPC of 0.01 (Table 1). Furthermore, while the proportion of children and adolescents with NP decreased relative to overall NP cases from 7.4% (1990) to 5.0% (2021) (Supplementary Figure S1), this declining trend was not noticeable when comparing prevalence rates during the same period (Supplementary Figure S2). The global incidence of NP among children and adolescents increased from 3.17 million cases in 1990 to 3.82 million cases in 2021. The age-standardized incidence rate showed a minimal decrease from 191.72 per 100,000 to 190.81 per 100,000, with an AAPC of −0.02% (Supplementary Table S1). The burden of NP in terms of YLDs increased from 884,574.61 in 1990 to 1,075,363.36 in 2021. However, the age-standardized YLD rate remained stable at approximately 53 per 100,000 over the study period, with an AAPC of 0% (Supplementary Table S1 and Supplementary Figure S3). These global trends suggest that while the overall age-standardized rates of NP have remained relatively stable, there are important variations by sex, age, and socioeconomic development.

**Table 1:** Age standardized prevalence and AAPC of NP in children and adolescents at global and regional level, 1990–2021.

Group/Region	Prevalence (95% UI)				
	No. of NP among children and adolescents in 1990	Age standardized rate in 1990 (per 100,000)	No. of NP among children and adolescents in 2021	Age standardized rate in 2021 (per 100,000)	AAPC (95% CI)
Global	8487242.95 (4639667.47 to 14218796.79)	512.15 (279.91 to 857.88)	10320495.67 (5623632.1 to 17369091.91)	513.37 (279.63 to 864.23)	0.01 (−0.01 to 0.02)
Sex					
Female	4690614.34 (2568460.86 to 7843929.15)	577 (315.88 to 964.71)	5750214.62 (3143678.1 to 9626556.35)	588.7 (321.72 to 985.88)	0.06 (0.05 to 0.08)
Male	3796628.61 (2,060,680 to 6435814.44)	449.7 (244.03 to 762.04)	4570281.05 (2474485.63 to 7759710.69)	442.19 (239.32 to 750.91)	−0.05 (−0.08 to −0.02)
Age group (years)					
5–9	557128.24 (268489.53 to 1055769.22)	95.48 (46.01 to 180.93)	669471.26 (322825.55 to 1263640.03)	97.44 (46.99 to 183.92)	0.1 (0.06 to 0.14)
10–14	2656335.33 (1455274.26 to 4318237.23)	495.88 (271.67 to 806.12)	3336182.1 (1835634.61 to 5413796.04)	500.45 (275.36 to 812.11)	0.03 (0.01 to 0.04)
15–19	5273779.38 (2915903.67 to 8844790.35)	1015.31 (561.37 to 1702.81)	6314842.31 (3465171.93 to 10691655.84)	1012.03 (555.33 to 1713.46)	−0.02 (−0.04 to 0.01)
SDI level					
High	1494651.99 (832223.92 to 2483849.38)	746.14 (415.15 to 1239.27)	1442657.62 (805248.39 to 2389118.5)	767.96 (428.36 to 1272.12)	0.09 (0.06 to 0.11)
High-middle	1596185.74 (877717.87 to 2677459.48)	540.66 (296.87 to 907.17)	1273029.62 (694114.6 to 2125580.36)	541.73 (295.33 to 904.67)	0 (−0.03 to 0.04)
Middle	2740000.18 (1473436.2 to 4640604.25)	467.73 (251.27 to 792.22)	2823140.29 (1520770.12 to 4768875.94)	482.04 (259.52 to 814.55)	0.09 (0.06 to 0.12)
Low-middle	1795548.1 (969461.37 to 3043770.35)	448.92 (242.67 to 760.62)	2712012.57 (1468901.97 to 4590343.37)	460.4 (249.18 to 779.6)	0.08 (0.03 to 0.13)
Low	852214.48 (462049.57 to 1442835.39)	485.6 (263.75 to 821.07)	2061227.62 (1119317.58 to 3483523.77)	500.9 (272.1 to 846.42)	0.1 (0.08 to 0.12)

AAPC, average annual percentage change; CI, confidence interval; SDI, sociodemographic index; NP, neck pain; UI, uncertainty interval.

### Global trends by sex

This analysis of NP among children and adolescents revealed distinct gender-specific patterns in global prevalence, incidence, and disability burden from 1990 to 2021. Females consistently exhibited higher rates of NP across all metrics compared to males (Table 1 and Supplementary Table S1). In 2021, the age-standardized prevalence rate was 588.70 per 100,000 for females versus 442.19 per 100,000 for males, representing a 33.1% higher burden among females. The age-standardized incidence rate in 2021 was 219.41 per 100,000 for females compared to 163.81 per 100,000 for males, showing a 33.9% higher rate in females. In 2021, females experienced 61.09 YLDs per 100,000 versus 46.31 per 100,000 for males, indicating a 31.9% higher disability burden among females.

From 1990 to 2021, the age-standardized prevalence, age-standardized incidence, and age-standardized YLDs of NP were higher in women than in men globally and across all SDI levels. In all SDI regions, these parameters increased in women and men except men in the high-middle SDI region (Supplementary Figures S4, S5).

### Global trends by age subgroup

This analysis revealed distinct age-specific patterns in NP burden among children and adolescents from 1990 to 2021 (Table 1; Supplementary Table S1; Supplementary Figure S6). A clear age gradient was observed, with prevalence rates in 2021 increasing from the 5–9 years group (97.44 per 100,000) to the 10–14 years group (500.45 per 100,000) and peaking in the 15–19 years group (1,012.03 per 100,000) (Table 1 and Supplementary Table S1). The temporal trends differed by age group: the 5–9 years group showed the largest increases in prevalence (AAPC: 0.10%), the 10–14 years group exhibited modest increases (AAPC: 0.03%), while the 15–19 years group demonstrated slight decreases (AAPC: −0.02%). Similar patterns were observed for incidence and YLDs, with the 15–19 age group experiencing approximately 10.4 times higher prevalence than the 5–9 age group and 2.0 times higher than the 10–14 age group in 2021. The absolute number of cases in 2021 was 669,471.26 for ages 5–9, 3,336,182.10 for ages 10–14, and 6,314,842.31 for ages 15–19 (Table 1 and Supplementary Table S1).

### Global trends by sociodemographic index

This analysis of NP among children and adolescents from 1990 to 2021 revealed notable variations across SDI levels (Table 1; Supplementary Table S1; Supplementary Figures S7–S9). In 2021, high SDI regions demonstrated the highest age-standardized prevalence rate at 767.96 per 100,000, followed by high-middle SDI (541.73), low SDI (500.90), middle SDI (482.04), and low-middle SDI regions (460.40). Temporal trends varied, with low SDI regions showing the largest prevalence increase (AAPC: 0.10%), while high-middle SDI regions remained stable (AAPC: 0%). Incidence rates in 2021 followed a similar pattern, with high SDI regions highest at 272.78 per 100,000, and all regions except high-middle showing increasing rates. YLDs in 2021 were also highest in high SDI regions (79.92 per 100,000), with the most substantial increases occurring in low SDI regions (AAPC: 0.12%). The distribution of absolute case numbers in 2021 reflected both population size and prevalence rates, with middle SDI regions carrying the largest burden (2,823,140.29 thousand cases), followed by low-middle SDI (2,712,012.57), low SDI (2,061,227.62), high SDI (1,442,657.62), and high-middle SDI regions (1,273,029.62).

### Regional trends

This analysis of NP in children and adolescents revealed substantial regional variations in 2021 (Supplementary Tables S2–S4 and Supplementary Figures S10, S11). The highest age-standardized prevalence rates were observed in Western Europe (984.25 per 100,000), High-income North America (827.69 per 100,000), and Central Europe (751.30 per 100,000), while the lowest rates were found in Southern Sub-Saharan Africa (384.20 per 100,000; 95% UI: 207.06–648.79), Oceania (390.60 per 100,000), and Central Sub-Saharan Africa (393.06 per 100,000). The largest increases in prevalence from 1990–2021 occurred in High-income North America, Southeast Asia, and Western Sub-Saharan Africa (all with AAPC of 0.13%), while the smallest changes were seen in Central Europe (AAPC: 0.02%), Eastern Europe (AAPC: 0.03%), and Southern Latin America (AAPC: 0.05%). For incidence, Western Europe (335.41 per 100,000), High-income North America (331.59 per 100,000), and High-income Asia Pacific (273.40 per 100,000) had the highest rates, whereas Southern Sub-Saharan Africa, Oceania, and Eastern Sub-Saharan Africa had the lowest. YLDs were highest in Western Europe (102.55 per 100,000), High-income North America (86.34 per 100,000), and Central Europe (78.26 per 100,000). In absolute terms, South Asia (2,194,835.53 thousand cases), East Asia (1,397,981.09 thousand), and Western Sub-Saharan Africa (1,075,064.70 thousand) carried the largest burden.

### National trends

**Figure 1:**
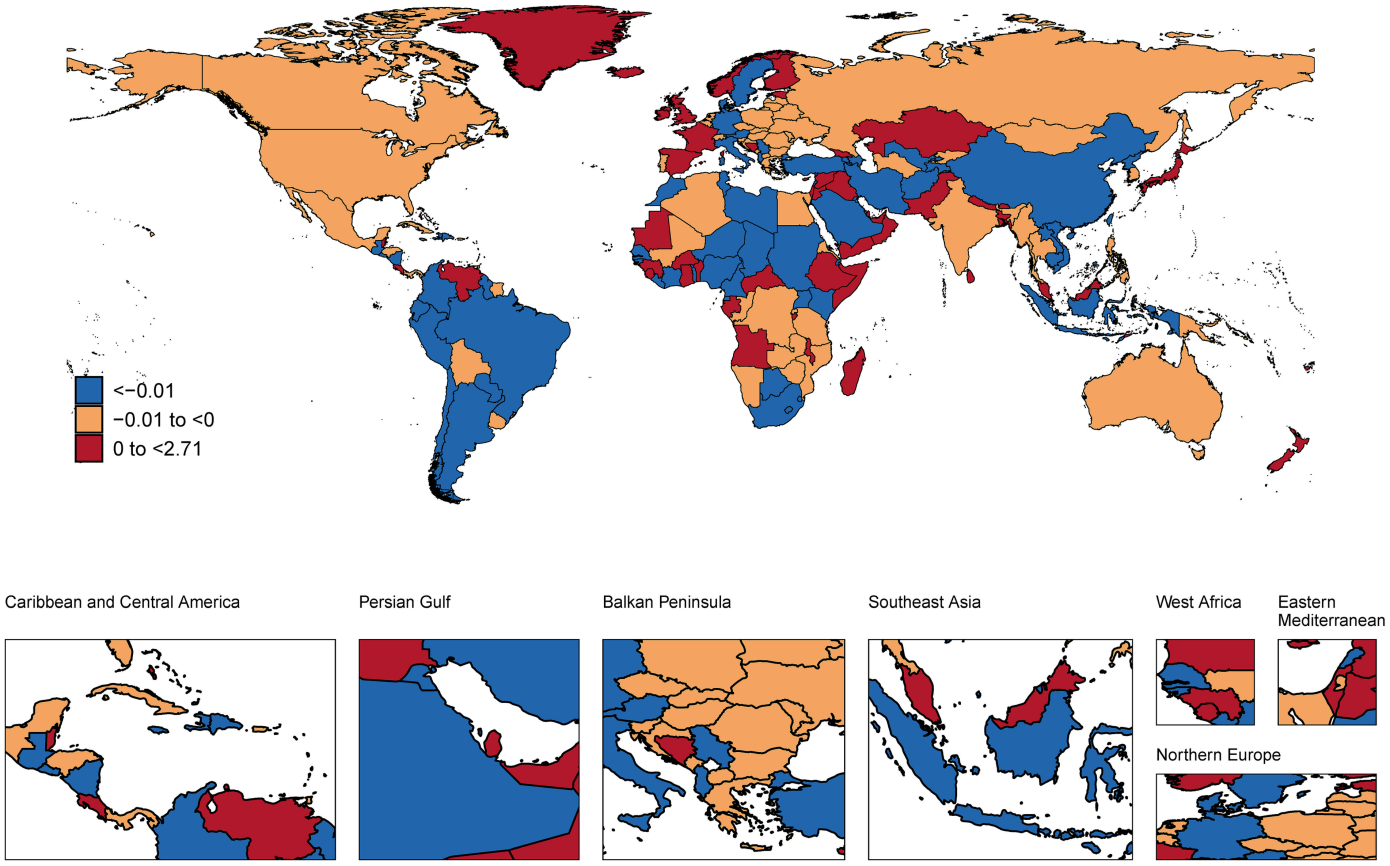
A map showing the AAPC in the prevalence of NP among children and adolescents in global populations, 1990–2021 (maps are used only to depict the GBD boundaries and not for administrative boundaries).

**Figure 2:**
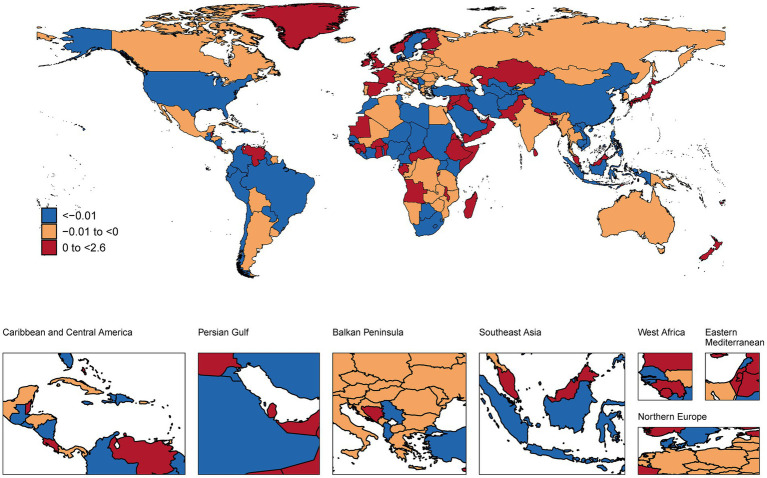
A map showing the AAPC in the incidence of NP among children and adolescents in global populations, 1990–2021 (maps are used only to depict the GBD boundaries and not for administrative boundaries).

**Figure 3:**
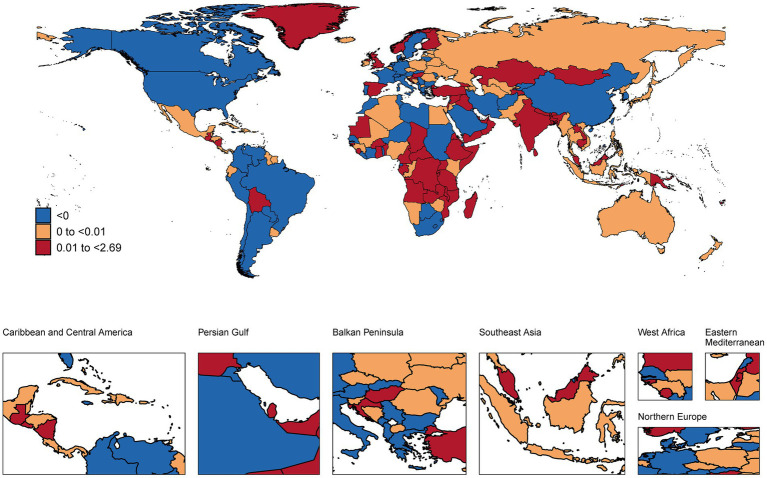
A map showing the AAPC in the YLDs of NP among children and adolescents in global populations, 1990–2021 (maps are used only to depict the GBD boundaries and not for administrative boundaries). AAPC, average annual percent change. GBD, Global Burden of Disease. NP, neck pain. YLDs, years lived with disability.

This analysis revealed substantial international variations in NP burden among children and adolescents (Figures 1–3 and Supplementary Table S5). In 2021, the highest age-standardized prevalence rates were observed in European countries, with Italy (1,082.81 per 100,000), Norway (1,061.86), and Denmark (1,060.10) leading, while the lowest rates occurred in Zimbabwe (302.57), Lesotho (310.05), and Eswatini (311.70). Temporal trends from 1990–2021 showed the largest increases in prevalence in Middle Eastern countries, with Qatar (AAPC: 0.50%), United Arab Emirates (0.39%), and Bahrain (0.34%) experiencing the most rapid rises, while Syria (−0.34%), Ukraine (−0.21%), and Lebanon (−0.17%) showed the steepest declines. Incidence patterns mirrored prevalence, with Sweden (362.95 per 100,000), United States (350.68), and Norway (350.66) having the highest rates, and Zimbabwe (110.56), Lesotho (113.22), and Eswatini (113.91) the lowest. YLDs were highest in Italy (112.88 per 100,000), Norway (110.69), and Denmark (110.66), and lowest in the same African nations. In absolute terms, the largest burdens were carried by populous countries, with India (1,714,474.33 thousand cases), China (1,300,979.00), and Nigeria (469,499.54) showing the highest total prevalence cases, while small island nations had the lowest absolute numbers.

## Discussion

To our knowledge, this is the most up-to-date study to describe the trends of NP disorders among children and adolescents at the global, regional, and national levels from 1990 to 2021. This study reveals several important patterns that warrant discussion. While our analysis documents substantial prevalence and incidence of NP, it is important to consider the functional impact of this condition through the lens of YLDs. The stable age-standardized YLD rate, despite increasing absolute numbers, suggests that many reported episodes of NP in this pediatric cohort, often acute or short-duration, may have a limited impact on overall disability. This is consistent with our case definition, which captures pain lasting at least 24 h but does not necessarily imply long-term functional limitation. However, the persistent 31.9% higher YLD burden among females compared to males underscores a significant gender disparity in the disabling impact of NP, warranting further investigation and targeted intervention. These findings provide critical insights for developing targeted prevention and intervention strategies for this increasingly recognized health concern.

### Age and sex disparities

The pronounced age gradient in NP prevalence, with rates increasing dramatically from the 5–9 years age group to the 15–19 years age group, aligns with previous research suggesting that NP becomes more common as children approach adolescence (18, 22). This pattern likely reflects the cumulative effects of several factors, including physical development, increased screen time, and academic pressures that intensify during adolescence (18, 23). Interestingly, while the 15–19 years group carries the highest burden, the 5–9 years group showed the largest relative increases over time, suggesting emerging concerns for younger children that merit attention in future research and interventions.

The consistently higher NP rates among females compared to males (33.1% higher prevalence in 2021) corroborate findings from adult populations and previous pediatric studies (24, 25). This gender disparity may reflect biological differences in musculoskeletal development, pain perception, and hormonal factors (26). Additionally, behavioral differences in physical activity patterns, study habits, and technology use between males and females may contribute to these disparities (27). Sex-specific prevention strategies may be necessary to effectively address these differences.

### Socioeconomic and regional patterns

The sociodemographic gradient revealed in our analysis, with high SDI regions demonstrating the highest age-standardized prevalence rates, contradicts the common assumption that musculoskeletal conditions primarily affect less developed regions (14). This pattern suggests that factors associated with higher socioeconomic development—such as increased sedentary behavior, technology use, and academic pressure—may contribute significantly to NP in children and adolescents (28, 29). However, the more rapid increases in prevalence observed in low SDI regions indicate a potential convergence of burden across development levels in the future, possibly reflecting global changes in lifestyle and technology access.

The substantial regional variations, with Western Europe, High-income North America, and Central Europe experiencing the highest prevalence rates, further support the association between socioeconomic development and NP burden. However, the notable differences between regions with similar development levels suggest that cultural, educational, and healthcare system factors may also play important roles (30). For instance, differences in school ergonomics, physical education requirements, and clinical awareness of pediatric NP may contribute to these variations (31).

### National trends and implications

At the national level, the predominance of European countries (Italy, Norway, and Denmark) among those with the highest prevalence rates underscores potential region-specific risk factors. The dramatic increases observed in rapidly developing Middle Eastern countries like Qatar and UAE (with AAPCs of 0.50 and 0.39%, respectively) may reflect rapid lifestyle transitions and increased technology adoption in these nations (32). Conversely, the decreases observed in countries experiencing conflict or socioeconomic challenges (Syria, Ukraine) might reflect either reduced reporting due to healthcare system disruptions or shifts in priorities away from musculoskeletal complaints during periods of crisis (33).

The absolute burden carried by populous countries like India, China, and Nigeria highlights the substantial public health impact of even moderate prevalence rates when applied to large populations. These findings suggest that while prevalence rates provide important insights, population-level burden estimates are essential for resource allocation and policy planning (34).

The interpretation of NP trends over time and the striking differences between neighboring countries with similar SDI (e.g., Norway vs. Sweden) require careful consideration of both true changes in disease burden and changes in awareness and reporting. In high-SDI countries, stable or slightly increasing trends might reflect a combination of genuine risk factor exposure and heightened clinical recognition of pediatric musculoskeletal pain (35, 36). Conversely, the rapid increases observed in many low-to-middle SDI regions could signal a true rise in NP due to lifestyle transitions, but also likely reflect improving healthcare access, diagnostic capabilities, and inclusion of NP in national health surveys (18). The substantial variations between neighboring countries suggest that factors beyond gross socioeconomic development, such as specific educational policies, school ergonomic standards, cultural attitudes toward pain reporting, and healthcare-seeking behaviors, may play a significant role in the recorded epidemiology of NP (18, 22). Disentangling the effects of true epidemiological change from surveillance artifact remains a challenge in cross-national burden studies.

### Clinical and public health implications

Despite the relatively stable global age-standardized rates of NP over the study period, the increasing absolute numbers and concerning trends in specific populations warrant attention from clinicians, researchers, and policymakers. The substantial burden among adolescents, particularly females, suggests the need for targeted screening and early intervention in these high-risk groups (37).

The higher rates in more developed regions point to potentially modifiable risk factors associated with modern lifestyle, such as prolonged device use and poor ergonomics (38, 39). Prevention strategies might include ergonomic education in schools, promotion of physical activity, and guidelines for technology use among children and adolescents (40, 41). The rising trends in younger children and in developing regions suggest a need for proactive approaches to prevent further increases as these populations adopt increasingly sedentary and technology-dependent lifestyles (42–45).

An intriguing finding of our study is the relative stability of age-standardized prevalence, incidence, and YLD rates for NP among children and adolescents from 1990 to 2021. This trend appears paradoxical, given the well-documented global increase in potential risk factors such as digital device use, sedentary behaviors, and psychosocial stressors during this period (46–48). Several factors might explain this apparent disconnect. First, improvements in public health awareness, ergonomics, and physical activity promotion in some regions may have mitigated the potential rise in NP burden. Second, the GBD case definition for NP, which requires pain lasting at least 24 h, may not fully capture the burden of transient, non-disabling pain episodes potentially linked to modern lifestyle factors. Finally, the modeling approach used in GBD, which smooths estimates over time and across data-sparse regions, might not detect subtle temporal shifts (18). This stability warrants cautious interpretation and highlights the need for longitudinal studies to clarify the relationship between contemporary risk factors and NP in young populations.

### Limitations and future directions

While our study provides insights, several limitations should be acknowledged. The GBD methodology relies on modeling approaches when primary data are limited, which may affect the precision of estimates, particularly for regions with sparse data (18, 22, 49). Additionally, the cross-sectional nature of most included studies limits causal inferences about risk factors for pediatric NP.

Future research should focus on longitudinal studies to better understand the natural history of NP in children, identify modifiable risk factors, and evaluate the effectiveness of preventive interventions. Studies examining the interactions between biological, psychological, and social factors in pediatric NP would also provide more comprehensive understanding of this condition (50).

While this study period extends to 2021, encompassing the COVID-19 pandemic, the GBD modeling approach smooths data and estimates trends over the entire period (18, 22). It is therefore not designed to detect or attribute short-term, acute changes resulting from specific events like pandemic-related lockdowns, which may have influenced risk factors such as screen time and physical activity levels (18, 22). Future studies specifically analyzing high-frequency data from this period are needed to elucidate the pandemic’s impact on pediatric NP.

## Conclusion

This study reveals that while age-standardized rates of NP among children and adolescents remain stable globally, the absolute burden continues to increase. The burden exhibits distinct patterns: females experience approximately 33% higher rates than males; prevalence increases dramatically with age, with the 15–19 age group showing 10-fold higher rates than the 5–9 group; and high-SDI regions demonstrate the highest prevalence, though low-SDI regions show the fastest growth. Furthermore, the analysis of YLDs highlights a significant disparity in the disabling impact of NP between females and males, emphasizing the need for interventions that address both the occurrence and the functional consequences of NP, tailored to gender, age, and socioeconomic context.

## Data Availability

The datasets presented in this study can be found in online repositories. The names of the repository/repositories and accession number(s) can be found in the article/Supplementary material.

## Footnotes

1
https://vizhub.healthdata.org/gbd-compare/

2
http://ghdx.healthdata.org/gbd-results-tool
