# Supplementary material for: Global, regional and national burden of neck pain in children and adolescents: GBD 2021 systematic analysis
Source: Front Neurol. 2025 Nov 24;16:1625954. doi: 10.3389/fneur.2025.1625954 (PMC12683374; doi:10.3389/fneur.2025.1625954)

Supplementary Table 1 | Age-standardized incidence and YLDs of NP in children and adolescents, and their AAPCs from 1990 to 2021 at the global and SDI levels.

	Incidence					YLDs				
	**Cases in 1990 (thousands)**	**Age-standardized rate in 1990 (per 100,000)**	**Cases in 2021 (thousands)**	**Age-standardized rate in 2021 (per 100,000)**	**AAPCs in rate, 1990-2021(%/year)**	**Cases in 1990 (thousands)**	**Age-standardized rate in 1990 (per 100,000)**	**Cases in 2021 (thousands)**	**Age-standardized rate in 2021 (per 100,000)**	**AAPCs in rate, 1990-2021(%/year)**
Global	3166665.34 (1512161.07 to 5992913.81)	191.72 (91.61 to 362.75)	3820012.77 (1823905.59 to 7160450.81)	190.81 (91.13 to 357.7)	-0.02 (-0.04 to 0.01)	884574.61 (413112.36 to 1640331.85)	53.38 (24.93 to 98.93)	1075363.36 (502637.55 to 1985032.14)	53.5 (25 to 98.76)	0 (-0.01 to 0.02)
Sex										
Female	1753700.02 (835674.78 to 3327704.64)	216.63 (103.31 to 410.95)	2133623.31 (1015143.93 to 4014218.79)	219.41 (104.43 to 412.87)	0.04 (0.02 to 0.06)	487089.04 (228063.5 to 899606.37)	59.93 (28.06 to 110.62)	596694.36 (281060.48 to 1101793.14)	61.09 (28.77 to 112.83)	0.06 (0.04 to 0.08)
Male	1412965.32 (674036.46 to 2670108.35)	167.77 (80.08 to 316.99)	1686389.46 (806171.74 to 3159976.88)	163.81 (78.31 to 306.95)	-0.08 (-0.1 to -0.05)	397485.57 (182463.52 to 738615.06)	47.08 (21.62 to 87.43)	478669 (221662.4 to 887140.81)	46.31 (21.44 to 85.84)	-0.06 (-0.09 to -0.02)
Age group (years)										

5-9	420929.13 (201197.2 to 815944.08)	72.13 (34.48 to 139.83)	505777.68 (241724.97 to 967881.4)	73.62 (35.18 to 140.87)	0.1 (0.06 to 0.14)	58871.07 (25208.93 to 117188.58)	10.09 (4.32 to 20.08)	70773.85 (31013.77 to 142138.55)	10.3 (4.51 to 20.69)	0.07 (0.03 to 0.11)
10-14	1043215.66 (517904.93 to 1933901.74)	194.75 (96.68 to 361.02)	1303743.55 (649522.18 to 2410678.7)	195.57 (97.43 to 361.62)	0.02 (-0.01 to 0.04)	277703.25 (131311.14 to 488277.59)	51.84 (24.51 to 91.15)	348740.12 (165452.28 to 617880.08)	52.31 (24.82 to 92.69)	0.03 (0.01 to 0.05)
15-19	1702520.55 (793058.94 to 3243067.99)	327.77 (152.68 to 624.36)	2010491.55 (932658.44 to 3781890.71)	322.2 (149.47 to 606.09)	-0.05 (-0.08 to -0.03)	548000.3 (256592.29 to 1034865.69)	105.5 (49.4 to 199.23)	655849.39 (306171.5 to 1225013.51)	105.11 (49.07 to 196.32)	-0.02 (-0.04 to 0)
SDI level										
High	521571.62 (255717.94 to 967123.59)	265.16 (130.4 to 491.06)	504590.19 (248676.73 to 926775.68)	272.78 (134.71 to 500.89)	0.09 (0.06 to 0.12)	156026.68 (74494.55 to 282637.58)	77.92 (37.21 to 140.82)	150065.94 (71749.66 to 269617.66)	79.92 (38.22 to 143.48)	0.07 (0.05 to 0.1)
High-middle	592231.29 (284811.18 to 1123389.58)	203.81 (98.17 to 386.51)	474780.7 (226859.54 to 886516.81)	202.37 (96.7 to 377.9)	-0.02 (-0.05 to 0)	167080.72 (78282.07 to 310563.63)	56.61 (26.53 to 105.02)	133271.93 (61907.2 to 245940.38)	56.71 (26.34 to 104.67)	0 (-0.04 to 0.05)

Middle	1057079.7 (495670.2 to 2029609.43)	182.03 (85.45 to 349.47)	1080650.86 (506962.29 to 2051112.14)	185.4 (87 to 351.98)	0.06 (0.03 to 0.08)	286292.83 (131248.84 to 540625.78)	48.88 (22.41 to 92.22)	295098.16 (136121.06 to 550427.42)	50.39 (23.24 to 94.01)	0.09 (0.06 to 0.13)
Low-middle	674351.89 (321947.18 to 1270291.64)	166.61 (79.47 to 313.88)	997727.22 (475791.5 to 1874761.84)	170.67 (81.42 to 320.7)	0.07 (0.01 to 0.13)	186350.87 (86818.88 to 345336.29)	46.58 (21.72 to 86.34)	282147.71 (132242.8 to 523506.45)	47.9 (22.45 to 88.91)	0.09 (0.04 to 0.14)
Low	318164.23 (151989.22 to 597235.46)	177.31 (84.48 to 332.85)	759090.2 (362264.76 to 1425261.09)	183.46 (87.46 to 344.5)	0.11 (0.09 to 0.13)	87922.66 (41095.82 to 162422.13)	50.08 (23.43 to 92.54)	213900.84 (99825.28 to 393543.89)	51.98 (24.25 to 95.67)	0.12 (0.1 to 0.14)

Note: Estimates are for Children and Adolescents. AAPCs=average annual percent changes. CI=confidence interval. P=P value for the significant test of AAPCs. SDI=Socio-demographic Index. YLDs=years lived with disability. NP=neck pain. Numbers in parentheses are 95% uncertainty intervals (Cases and age-standardized rate) and 95% confidence interval (AAPCs).

Supplementary Table 2 | Age-standardized prevalence of NP in children and adolescents, and their AAPCs from 1990 to 2021 at regional levels.

Regions	Prevalence				
	**Cases in 1990 (thousands)**	**Age-standardized rate in 1990 (per 100,000)**	**Cases in 2021 (thousands)**	**Age-standardized rate in 2021 (per 100,000)**	**AAPCs in rate, 1990-2021 (%/year)**
Andean Latin America	89732.5<br>(48151.49 to 151893.14)	663.54<br>(356.13 to 1123.27)	118206.48<br>(63489.57 to 200365.27)	661.24<br>(355 to 1121.01)	-0.01<br>(-0.01 to -0.01)
Australasia	25681.52<br>(13982.97 to 44093.28)	511.98<br>(278.38 to 877.75)	29888.37<br>(16261.82 to 51215.9)	511.72<br>(278.24 to 877.32)	-0.01<br>(-0.03 to 0.02)
Caribbean	76259.17<br>(40970.66 to 129091.55)	663.61<br>(356.19 to 1123.45)	78573.67<br>(42214.07 to 133057.65)	662.67<br>(355.71 to 1122.45)	0<br>(-0.01 to 0)
Central Asia	139292.48<br>(74366.05 to 235390.27)	645.26<br>(344.67 to 1089.84)	151248.81<br>(80707.2 to 255673.83)	644.42<br>(344.24 to 1088.44)	0<br>(0 to 0)
Central Europe	211939.06<br>(113722.27 to 355960.35)	674.07<br>(361.35 to 1133.41)	126980.49<br>(68193.48 to 213207.71)	675.14<br>(362.2 to 1134.97)	0<br>(0 to 0.01)

Central Latin America	431516.49 (231664.28 to 729797.93)	723.54 (388.41 to 1223.81)	493679.62 (265370.57 to 834925.7)	718.75 (385.98 to 1216.71)	-0.02 (-0.02 to -0.02)
Central Sub-Saharan Africa	108990.17 (58213.64 to 186097.26)	562.63 (300.91 to 959.8)	283582.54 (151545.17 to 483813.91)	562.55 (300.85 to 959.66)	0 (0 to 0)
East Asia	1395618.09 (730518.81 to 2386018.91)	363.42 (189.68 to 622.89)	931060.62 (500942.32 to 1600694.07)	361.15 (194.45 to 620.71)	-0.02 (-0.02 to -0.02)
Eastern Europe	370781.66 (201732.06 to 623894.24)	731.48 (397.89 to 1230.91)	261026.45 (142020.62 to 439048.87)	733.02 (398.87 to 1233.13)	0.01 (0.01 to 0.01)
Eastern Sub-Saharan Africa	307759.32 (165883.19 to 518130.38)	436.82 (235.78 to 734.53)	707298.54 (381581.74 to 1188594.29)	436.22 (235.38 to 732.98)	0 (-0.02 to 0.02)
High-income Asia Pacific	333487.62 (181083.34 to 561412.94)	745.44 (404.82 to 1253.51)	193830.44 (105250.1 to 326214.27)	748.21 (406.19 to 1258.9)	0.01 (0.01 to 0.02)

High-income North America	493164.64 (269478.94 to 835437.81)	789.48 (431.16 to 1337.15)	585428.25 (319909.59 to 990802.97)	790.3 (431.35 to 1338.44)	0 (-0.05 to 0.05)
North Africa and Middle East	1539767.47 (843868.88 to 2556572.5)	634.67 (348.1 to 1053.57)	2176252.42 (1192591.7 to 3611727.11)	624.16 (342.07 to 1036.02)	-0.05 (-0.07 to -0.04)
Oceania	8482.84 (4520.14 to 14525.69)	370.92 (197.82 to 634.72)	16030.81 (8543.67 to 27436.85)	370.69 (197.72 to 634.04)	0 (0 to 0)
South Asia	2708775.48 (1452971.64 to 4654533.49)	368.22 (197.79 to 632.34)	4075301.82 (2191367.44 to 6996312.55)	368.76 (197.98 to 633.46)	0.01 (-0.06 to 0.08)
South-East Asia Region	605147.55 (322922.53 to 1033229.67)	375.79 (200.57 to 641.63)	681442.73 (363767.12 to 1162835.87)	379.1 (202.16 to 647.49)	0.03 (0.03 to 0.03)
Southern Latin America	100408.63 (55135.79 to 170417.68)	698.3 (383.4 to 1185.42)	109850.28 (60293.74 to 186473.81)	696.99 (382.55 to 1183.46)	-0.01 (-0.01 to -0.01)

Southern Sub-Saharan Africa	118305.07 (64378.44 to 202949.85)	627.68 (341.63 to 1076.76)	145610.13 (79144.95 to 249882.35)	621.67 (337.82 to 1067.18)	-0.03 (-0.03 to -0.03)
Tropical Latin America	382186.32 (202374.68 to 649489.22)	739.64 (391.71 to 1256.72)	380377.02 (201699.36 to 645462.65)	736.83 (390.39 to 1251.43)	-0.02 (-0.02 to -0.01)
Western Europe	708301.61 (406564.98 to 1186504.2)	874.06 (500.55 to 1462.43)	670353.2 (379746.88 to 1117748.33)	909.29 (514.54 to 1515.99)	0.13 (0.1 to 0.16)
Western Sub-Saharan Africa	455916.74 (248586.32 to 769977.66)	680.29 (371.69 to 1147.11)	1230250.1 (671302.22 to 2076537.83)	678.92 (370.9 to 1145.11)	-0.01 (-0.01 to 0)

Note: Estimates are for Children and Adolescents. AAPCs=average annual percent changes. CI=confidence interval. NP=low neck pain. P=P value for the significant test of AAPCs. SDI=Socio-demographic Index. Numbers in parentheses are 95% uncertainty intervals (Cases and age standardized rate) and 95% confidence interval (AAPCs).

Supplementary Table 3 | Age-standardized incidence and YLDs of NP in children and adolescents, and their AAPCs from 1990 to 2021 at the regional levels.

Regions	Incidence					YLDs				
	**Cases in 1990 (thousands)**	**Age-standardized rate in 1990 (per 100,000)**	**Cases in 2021 (thousands)**	**Age-standardized rate in 2021 (per 100,000)**	**AAPCs in rate, 1990-2021 (%/year)**	**Cases in 1990 (thousands)**	**Age-standardized rate in 1990 (per 100,000)**	**Cases in 2021 (thousands)**	**Age-standardized rate in 2021 (per 100,000)**	**AAPCs in rate, 1990-2021 (%/year)**
Andean Latin America	34671.16 (16381.09 to 64943.55)	255.7 (120.75 to 478.75)	45368.33 (21416.03 to 84937.68)	254.89 (120.37 to 477.46)	-0.01 (-0.01 to -0.01)	9356.11 (4266.38 to 17244.16)	69.18 (31.56 to 127.54)	12315.43 (5555.88 to 22937.37)	68.9 (31.07 to 128.3)	-0.01 (-0.03 to 0)
Australasia	8338.27 (4045.05 to 15698.14)	171.14 (83.57 to 321.28)	9907.4 (4837.13 to 18597.71)	171.06 (83.54 to 321.19)	0 (-0.02 to 0.02)	2676.97 (1235.67 to 5073.11)	53.43 (24.61 to 100.92)	3110.12 (1407.77 to 5798.91)	53.26 (24.08 to 99.34)	0 (-0.06 to 0.05)
Caribbean	29088.03 (13716.26 to 54374.4)	255.74 (120.77 to 478.78)	30026.6 (14166.29 to 56159.35)	255.4 (120.61 to 478.22)	0 (0 to 0)	7913.34 (3585.65 to 14632.02)	68.88 (31.22 to 127.25)	8160.8 (3657.3 to 15043.35)	68.84 (30.86 to 126.85)	-0.01 (-0.01 to 0)
Central Asia	53178.38 (25451.19 to 99056.22)	245.21 (117.36 to 456.59)	58152.52 (27847.88 to 108310.86)	244.89 (117.2 to 455.9)	0 (0 to 0)	14534.55 (6762.33 to 26966.09)	67.33 (31.34 to 124.89)	15798.51 (7356.22 to 29177.45)	67.3 (31.33 to 124.38)	0 (-0.02 to 0.01)
Central Europe	80646.63	258.61	48316.06	259.14	0.01	22166.42 (10229.98	70.51	13284.26	70.64	0.01

	(38699.64 to 149737.06)	(124.06 to 480.48)	(23193.51 to 89744.94)	(124.35 to 481.69)	(0 to 0.01)	to 41309.53)	(32.51 to 131.47)	(6189.62 to 25060.11)	(32.89 to 133.34)	(-0.01 to 0.02)
Central Latin America	169160.71 (80388.67 to 323240.06)	283.56 (134.75 to 541.77)	191455.24 (90920.01 to 364842.58)	281.56 (133.71 to 537.31)	-0.02 (-0.03 to -0.02)	45083.56 (20607.48 to 83415.72)	75.59 (34.55 to 139.89)	51607.92 (23550.77 to 96056.05)	75.16 (34.27 to 139.91)	-0.02 (-0.02 to -0.01)
Central Sub-Saharan Africa	40694.88 (19573.7 to 78512.98)	206.37 (99.11 to 398.6)	105277.04 (50633.11 to 203126.18)	206.32 (99.08 to 398.52)	0 (0 to 0)	11168.78 (5081.01 to 20987.77)	57.64 (26.29 to 108.38)	29384.18 (13034.74 to 54238.05)	58.27 (25.9 to 107.65)	0.02 (0 to 0.05)
East Asia	541045.06 (252329.83 to 1063357.11)	143.83 (67.3 to 282.59)	369515.07 (171093.88 to 698011.09)	142.56 (65.96 to 269.14)	-0.03 (-0.03 to -0.02)	146858.54 (65971.39 to 278968.45)	38.26 (17.18 to 72.58)	98234.67 (43907.55 to 184662.95)	38.1 (17.03 to 71.63)	-0.01 (-0.02 to 0)
Eastern Europe	144002 (68421.76 to 267860.67)	285 (135.42 to 530.22)	102129.74 (48502.28 to 190026.22)	285.73 (135.69 to 531.6)	0.01 (0.01 to 0.01)	38785.97 (17945.76 to 72700.13)	76.52 (35.41 to 143.41)	27336.38 (12765.26 to 51112.88)	76.76 (35.84 to 143.61)	0.01 (0 to 0.02)
Eastern Sub-Saharan Africa	113716.15 (54721.91 to 211236.02)	158.22 (75.85 to 293.85)	256866.67 (123353.01	157.88 (75.74 to 292.88)	-0.01 (-0.03 to 0.02)	31856.99 (14864.62	45.2 (21.1 to 82.85)	73623.19 (33776.93	45.4 (20.83 to 83.41)	0.01 (-0.02 to 0.05)

			to 476504.95)			to 58340.44)		to 135209.18)		
High-income Asia Pacific	116733.61 (57491.33 to 219892.99)	269.69 (133.13 to 506.61)	68895.65 (33950.76 to 129580.28)	270.93 (133.65 to 508.9)	0.02 (0 to 0.03)	34889.81 (16337.47 to 63949.45)	78.03 (36.56 to 142.68)	20293.12 (9575.07 to 37240.8)	78.36 (36.98 to 143.6)	0.02 (-0.01 to 0.04)
High-income North America	180501.51 (87319.78 to 335284.37)	292.19 (141.53 to 543.27)	212824.62 (102896.43 to 394999.19)	292.54 (141.65 to 543.86)	-0.01 (-0.05 to 0.04)	51402.65 (24383.68 to 94663.43)	82.32 (39.04 to 151.4)	60661.17 (28724.66 to 110590.82)	81.94 (38.77 to 149.25)	-0.01 (-0.08 to 0.05)
North Africa and Middle East	573825.87 (276618.48 to 1069326.27)	234.24 (112.83 to 436.8)	802584.64 (387086.95 to 1493950.51)	229.76 (110.78 to 427.77)	-0.07 (-0.08 to -0.05)	160073.49 (75687.17 to 293369.1)	65.97 (31.21 to 121.01)	226366.63 (106156.65 to 413738.12)	64.92 (30.44 to 118.7)	-0.05 (-0.07 to -0.03)
Oceania	3360.08 (1576.72 to 6481.71)	145.99 (68.43 to 281.34)	6343.87 (2976.65 to 12234.56)	145.86 (68.38 to 281.09)	0 (0 to 0)	883.23 (402.8 to 1648.24)	38.61 (17.6 to 72.01)	1680.7 (749.2 to 3209.24)	38.85 (17.33 to 74.19)	0.02 (0.01 to 0.03)
South Asia	996991.73 (480719.64 to 1873350.25)	133.55 (64.33 to 250.9)	1455818.06 (700021.87 to 2734243.38)	133.8 (64.41 to 251.35)	0.01 (-0.09 to 0.11)	280649.43 (129987.13 to 518032.05)	38.14 (17.69 to 70.35)	423723.11 (197908.3 to 780547.77)	38.35 (17.9 to 70.72)	0.02 (-0.04 to 0.09)

South-East Asia Region	237929.73 (111437.22 to 464660.27)	147.62 (69.12 to 288.3)	265953.25 (124448.52 to 520434.09)	149.05 (69.77 to 291.73)	0.03 (0.03 to 0.03)	63323.95 (28686.88 to 119436.36)	39.32 (17.81 to 74.19)	71454.92 (32943.6 to 135219.6)	39.76 (18.32 to 75.27)	0.04 (0.03 to 0.05)
Southern Latin America	35599.71 (17346.96 to 67111.07)	248.38 (121.06 to 468.29)	38651.16 (18803.68 to 72865.14)	248.02 (120.84 to 467.71)	0 (-0.01 to 0)	10478.84 (4790.36 to 19433.17)	72.88 (33.32 to 135.18)	11430.59 (5399.42 to 20987.18)	72.54 (34.26 to 133.17)	-0.02 (-0.03 to 0)
Southern Sub-Saharan Africa	44515.76 (21323.93 to 85595.48)	235.75 (112.92 to 453.3)	54559.74 (26174.96 to 104920.47)	233.31 (111.91 to 448.66)	-0.03 (-0.04 to -0.03)	12305.58 (5779.73 to 22786.29)	65.29 (30.67 to 120.9)	15143.68 (7123.49 to 28419.92)	64.65 (30.41 to 121.38)	-0.03 (-0.04 to -0.01)
Tropical Latin America	152624.5 (71199.39 to 296065.13)	294.66 (137.42 to 571.59)	150227.14 (70017.18 to 291370.67)	293.45 (136.87 to 569.2)	-0.02 (-0.02 to -0.01)	39662.4 (18290.91 to 75105.24)	76.75 (35.4 to 145.44)	39456.9 (17996.96 to 74837.19)	76.45 (34.83 to 144.92)	-0.02 (-0.03 to 0)
Western Europe	223511.86 (113206.89 to 415282.06)	285.65 (145.69 to 529.41)	215433.5 (108763.33 to 402066.11)	298.17 (150.96 to 555.95)	0.14 (0.1 to 0.18)	73833.94 (36026.16 to 131197.97)	91.18 (44.53 to 161.55)	69735.83 (33681.21 to 124072.4)	94.63 (45.73 to 168.24)	0.12 (0.08 to 0.16)
Western Sub-Saharan Africa	171938.51	251.89	460907.85 (221363.64	251.56	0	47031.54 (22232.54	70.16	127606.11 (60086.47	70.41	0.01

	(82613.28 to 324124.93)	(120.86 to 474.3)	to 868605.57)	(120.68 to 473.78)	(-0.01 to 0)	to 87781.48)	(33.2 to 131.06)	to 238656.36)	(33.16 to 131.77)	(0 to 0.02)

Note: Estimates are for Children and Adolescents. AAPCs=average annual percent changes. CI=confidence interval. P=P value for the significant test of AAPCs. YLDs=years lived with disability. NP=neck pain. Numbers in parentheses are 95% uncertainty intervals (Cases and age-standardized rate) and 95% confidence interval (AAPCs).

Supplementary Table 4 | Age-standardized prevalence, incidence and YLDs of NP in children and adolescents in 2021 at regional levels by sex.

**Regions**	**Age-standardized rate in 2021 (per 100,000)**					
	**Male**			**Female**		
	**Prevalence**	**Incidence**	**YLDs**	**Prevalence**	**Incidence**	**YLDs**
Andean Latin America	602.16 (324.94 to 1026.57)	232.97 (111.26 to 437.95)	62.97 (28.35 to 117.23)	724.76 (386.21 to 1225.57)	278.43 (130.88 to 530.85)	75.27 (33.75 to 139.17)
Australasia	415.29 (221.2 to 715.12)	141.25 (67.63 to 272.05)	43.48 (18.44 to 83.91)	613.91 (334.95 to 1047.01)	202.64 (99.29 to 375.28)	63.62 (28.5 to 118.64)
Caribbean	602.16 (324.94 to 1026.57)	232.97 (111.26 to 437.95)	62.87 (28.58 to 114.86)	724.76 (386.21 to 1225.57)	278.43 (130.88 to 530.85)	74.97 (34.11 to 140.08)
Central Asia	591.77 (317.66 to 1003.56)	226.12 (107.53 to 431.81)	62.12 (28.35 to 115.07)	700.49 (372.08 to 1194.42)	264.91 (126.28 to 505.96)	72.83 (34.01 to 133.64)
Central Europe	621.91 (335.68 to 1055.58)	239.5 (114.11 to 453.01)	65.25 (30.54 to 123.49)	731.71 (388.41 to 1240.32)	280.03 (134.17 to 523.45)	76.37 (35.36 to 142.12)

Central Latin America	654.62 (348.61 to 1121.22)	256.77 (121.43 to 487.07)	68.73 (30.6 to 128.8)	784.36 (422.01 to 1324.84)	306.97 (145.56 to 586.94)	81.74 (37.92 to 152.63)
Central Sub-Saharan Africa	474.54 (253.78 to 816.99)	173.26 (83.18 to 332.54)	49.38 (21.4 to 92.96)	651.76 (351.51 to 1099.87)	239.85 (115.15 to 459.2)	67.28 (30.5 to 124.16)
East Asia	350.42 (186.48 to 602.23)	135.22 (63.37 to 258.24)	37.03 (16.48 to 69.59)	373.57 (198 to 637.44)	151.05 (69.18 to 290.63)	39.33 (17.39 to 74.38)
Eastern Europe	678.95 (364.58 to 1157.44)	264.6 (124.41 to 496.09)	71.38 (32.72 to 137.41)	789.99 (432.76 to 1330.63)	307.99 (149.08 to 580.55)	82.42 (38.53 to 153.05)
Eastern Sub-Saharan Africa	399.69 (214.74 to 677.31)	142.49 (68.79 to 262.3)	41.67 (19.07 to 76.46)	473.01 (253.42 to 810.92)	173.4 (82.32 to 326.85)	49.17 (22.31 to 90.82)
High-income Asia Pacific	671.43 (364.25 to 1119.47)	245.82 (121.34 to 465.71)	70.46 (33.26 to 130.12)	829.63 (452.33 to 1403.07)	297.52 (145.89 to 561.56)	86.73 (40.83 to 158.48)

High-income North America	664.01 (356.53 to 1140.85)	249.1 (119.99 to 470.9)	69.2 (31.87 to 125.18)	922.5 (508.55 to 1549.47)	337.99 (164.29 to 632.37)	95.28 (44.98 to 177.15)
North Africa and Middle East	439.76 (241.2 to 732.67)	154.04 (74.94 to 280.78)	45.98 (21.63 to 85.28)	821.25 (446 to 1379.93)	310.68 (148.18 to 590.83)	85.16 (39.75 to 156.83)
Oceania	357.07 (187.64 to 605.32)	137.88 (65.24 to 265.94)	37.44 (16.25 to 73.46)	385.76 (204.57 to 668.87)	154.69 (71.31 to 300.08)	40.42 (18.29 to 76.28)
South Asia	335.05 (179.97 to 573.61)	121.33 (58.69 to 224.84)	35.05 (15.79 to 64.64)	405.09 (219.13 to 692.52)	147.27 (70.93 to 283.43)	41.9 (20.12 to 76.84)
Southeast Asia	357.4 (190.11 to 614.29)	138.18 (64.55 to 269.58)	37.63 (17.13 to 71.23)	401.9 (213.9 to 684.12)	160.48 (74.97 to 313.26)	41.99 (19.42 to 79.28)
Southern Latin America	599.03 (319.59 to 1026.87)	218.81 (104.87 to 413.68)	62.69 (28.98 to 118.04)	798.41 (439.51 to 1343.57)	278.31 (137.69 to 518.77)	82.75 (38.14 to 150.79)

Southern Sub-Saharan Africa	525.2 (284.3 to 904.67)	195.67 (93.96 to 375.9)	54.95 (25.64 to 103.85)	719.32 (394.47 to 1230.05)	271.43 (128.62 to 521.69)	74.48 (34.82 to 138.68)
Tropical Latin America	663.27 (352.48 to 1148.78)	263.72 (123.54 to 510.46)	69.38 (31.45 to 134.47)	812.9 (434.97 to 1370.15)	324.21 (149.66 to 635.25)	83.76 (38.05 to 158.99)
Western Europe	733.04 (407.26 to 1244.46)	252.6 (125.68 to 474.72)	76.78 (36.15 to 139.57)	1095.76 (626.55 to 1811.17)	346.29 (176.6 to 639.83)	113.51 (55.53 to 198.51)
Western Sub-Saharan Africa	511.05 (275.95 to 881.85)	189.45 (91.48 to 364.07)	53.34 (25.01 to 100.19)	840.5 (460.01 to 1415.22)	311.65 (149.11 to 581.27)	86.85 (40.52 to 161.07)

Note: Estimates are for Children and Adolescents. AAPCs=average annual percent changes. CI=confidence interval. YLDs=years lived with disability. NP=neck pain. P=P value for the significant test of AAPCs. Numbers in parentheses are 95% uncertainty intervals.

Supplementary Table 5 | Age-standardized prevalence, incidence and YLDs of NP in children and adolescents, and their AAPCs from 1990 to 2021 at the regional levels.

	**Age-standardized rate in 2021 (per 100,000)**			**AAPCs 1990-2021 (%/year)**		
	**Prevalence**	**Incidence**	**YLDs**	**Prevalence**	**Incidence**	**YLDs**
Afghanistan	608.93<br>(333.33 to 1012.76)	223.16<br>(107.73 to 412.39)	63.12<br>(28.21 to 115.96)	-0.04<br>(-0.07 to -0.01)	-0.04<br>(-0.07 to -0.02)	-0.03<br>(-0.08 to 0.02)
Albania	643.37<br>(343.71 to 1086.39)	244.53<br>(117.01 to 455.28)	67.12<br>(30.8 to 125.61)	-0.01<br>(-0.01 to -0.01)	-0.01<br>(-0.01 to -0.01)	-0.02<br>(-0.04 to 0.01)
Algeria	610.64<br>(334.27 to 1015.36)	223.85<br>(108.02 to 413.86)	63.74<br>(29.28 to 117.21)	0<br>(0 to 0)	0<br>(0 to 0)	0<br>(-0.01 to 0.01)
American Samoa	370.82<br>(197.76 to 634.48)	145.94<br>(68.41 to 281.26)	38.83<br>(17.38 to 72.1)	0<br>(0 to 0)	0<br>(0 to 0)	0.01<br>(0 to 0.02)
Andorra	912.71<br>(516.93 to 1523.33)	291.16<br>(146.59 to 541.87)	94.86<br>(44.82 to 170.32)	0.01<br>(0 to 0.02)	0<br>(0 to 0.01)	-0.01<br>(-0.02 to 0.01)
Angola	564.77<br>(302.11 to 962.52)	207.13<br>(99.48 to 400.14)	58.66	0.01<br>(0.01 to 0.02)	0.01<br>(0.01 to 0.01)	0.03

			(26.48 to 110.12)			(0.02 to 0.05)
Antigua and Barbuda	662.21	255.25	69.12	0	0	-0.01
	(355.5 to 1122.06)	(120.54 to 477.99)	(30.02 to 128.19)	(-0.01 to 0)	(-0.01 to 0)	(-0.03 to 0)
Argentina	697.32	248.11	72.61	-0.01	0	-0.02
	(382.77 to 1183.95)	(120.89 to 467.85)	(33.54 to 134.09)	(-0.01 to -0.01)	(0 to 0)	(-0.04 to 0.01)
Armenia	642.68	244.28	67.08	-0.01	-0.01	0.01
	(343.35 to 1084.96)	(116.87 to 454.92)	(30.27 to 123.88)	(-0.01 to -0.01)	(-0.01 to -0.01)	(-0.01 to 0.02)
Australia	547.32	181.1	56.95	0	0	0
	(298.32 to 937.93)	(88.63 to 336.01)	(25.53 to 105.51)	(0 to 0)	(0 to 0)	(-0.01 to 0.01)
Austria	1139.82	353.63	118.74	-0.01	0	-0.01
	(646.96 to 1872.24)	(176.65 to 655.92)	(57.08 to 214.38)	(-0.03 to 0.01)	(-0.01 to 0)	(-0.07 to 0.05)
Azerbaijan	642.52	244.21	67.21	-0.01	-0.01	0
	(343.27 to 1084.62)	(116.83 to 454.82)	(30.11 to 125.03)	(-0.01 to -0.01)	(-0.01 to -0.01)	(-0.01 to 0.02)

Bahamas	663.84 (356.31 to 1123.7)	255.83 (120.81 to 478.95)	69.09 (31.04 to 126.85)	0 (0 to 0)	0 (0 to 0)	0.01 (-0.02 to 0.03)
Bahrain	594.57 (325.15 to 986.95)	217.92 (105.3 to 402.14)	62.06 (29.07 to 115.98)	-0.12 (-0.18 to -0.05)	-0.12 (-0.18 to -0.05)	-0.11 (-0.18 to -0.04)
Bangladesh	324.5 (173.79 to 551.9)	114.52 (55.94 to 218.13)	33.86 (14.86 to 63.23)	0 (0 to 0)	0 (0 to 0)	0.02 (0.01 to 0.04)
Barbados	662.06 (355.4 to 1121.79)	255.17 (120.51 to 477.88)	69.15 (32.55 to 127.48)	0 (0 to 0)	0 (0 to 0)	0.01 (-0.04 to 0.05)
Belarus	644.48 (344.27 to 1088.56)	244.93 (117.22 to 455.94)	67.59 (30.37 to 123.63)	0 (0 to 0)	0 (0 to 0)	0 (-0.01 to 0.01)
Belgium	918.43 (518.05 to 1526.77)	294.18 (147.21 to 544.75)	95.86 (45.8 to 171.88)	0 (-0.05 to 0.04)	0 (-0.04 to 0.04)	0 (-0.05 to 0.05)

Belize	663.54<br>(356.15 to 1123.35)	255.71<br>(120.76 to 478.74)	69.09<br>(31.05 to 125.82)	0<br>(0 to 0)	0<br>(0 to 0)	0<br>(0 to 0.01)
Benin	631.92<br>(343.49 to 1074.28)	229.32<br>(109.15 to 436.57)	65.7<br>(29.66 to 124.68)	0.01<br>(0 to 0.01)	0.01<br>(0.01 to 0.01)	0.02<br>(0.01 to 0.03)
Bermuda	662.66<br>(355.71 to 1122.48)	255.4<br>(120.61 to 478.24)	69.41<br>(30.13 to 127.75)	0<br>(0 to 0)	0<br>(0 to 0)	0.01<br>(-0.04 to 0.07)
Bhutan	323.81<br>(173.49 to 551.49)	114.31<br>(55.79 to 217.61)	33.89<br>(15.02 to 63.88)	0.02<br>(0.02 to 0.03)	0.02<br>(0.02 to 0.03)	0.03<br>(0.02 to 0.04)
Bolivia (Plurinational State of)	662.72<br>(355.74 to 1122.48)	255.41<br>(120.62 to 478.24)	69.12<br>(30.8 to 127.57)	0<br>(0 to 0)	0<br>(0 to 0)	0.01<br>(0 to 0.02)
Bosnia and Herzegovina	644.68<br>(344.37 to 1088.87)	244.99<br>(117.25 to 456.08)	67.52<br>(30.22 to 128.73)	0<br>(0 to 0)	0<br>(0 to 0)	0.01<br>(0 to 0.02)

Botswana	562.33<br>(300.72 to 959.37)	206.23<br>(99.03 to 398.35)	58.64<br>(27.09 to 109.04)	-0.02<br>(-0.02 to -0.02)	-0.02<br>(-0.02 to -0.02)	-0.01<br>(-0.02 to 0)
Brazil	740.83<br>(392.31 to 1260.23)	295.36<br>(137.83 to 572.48)	76.86<br>(35 to 146.11)	-0.01<br>(-0.02 to 0)	-0.01<br>(-0.02 to 0)	-0.01<br>(-0.03 to 0.01)
Brunei Darussalam	655.93<br>(356.62 to 1076.28)	230.01<br>(114.06 to 425.01)	68.75<br>(32.09 to 124.26)	-0.02<br>(-0.02 to -0.01)	-0.01<br>(-0.02 to -0.01)	-0.01<br>(-0.03 to 0)
Bulgaria	644.47<br>(344.27 to 1088.54)	244.92<br>(117.22 to 455.93)	67.33<br>(30.76 to 125.94)	0<br>(0 to 0)	0<br>(0 to 0)	-0.01<br>(-0.02 to 0.01)
Burkina Faso	634.06<br>(344.68 to 1077.9)	230.06<br>(109.48 to 437.75)	65.76<br>(29.06 to 123.46)	0.02<br>(0.01 to 0.02)	0.02<br>(0.01 to 0.02)	0.04<br>(0.04 to 0.05)
Burundi	410.53<br>(220.81 to 689.92)	146.8<br>(70.59 to 270.47)	42.66<br>(18.87 to 79.94)	0<br>(0 to 0.01)	0<br>(0 to 0.01)	0.03<br>(0.02 to 0.04)

Cabo Verde	628.93<br>(341.81 to 1068.93)	228.4<br>(108.8 to 435.26)	65.71<br>(29.88 to 123.09)	-0.01<br>(-0.01 to -0.01)	-0.01<br>(-0.01 to -0.01)	0<br>(-0.01 to 0.01)
Cambodia	342.19<br>(183.74 to 578.25)	132.65<br>(61.07 to 254.69)	35.93<br>(15.36 to 68.13)	-0.01<br>(-0.01 to -0.01)	-0.01<br>(-0.01 to -0.01)	0.02<br>(0.01 to 0.03)
Cameroon	629.27<br>(342.01 to 1069.68)	228.44<br>(108.79 to 435.24)	65.45<br>(30.03 to 124.09)	-0.01<br>(-0.01 to -0.01)	-0.01<br>(-0.01 to -0.01)	0.02<br>(-0.01 to 0.04)
Canada	694.62<br>(384.68 to 1184.23)	247.74<br>(119.37 to 467.31)	72.56<br>(34.22 to 131.2)	0<br>(0 to 0)	0<br>(0 to 0)	-0.01<br>(-0.01 to 0)
Central African Republic	563.58<br>(301.41 to 960.94)	206.66<br>(99.24 to 399.23)	58.27<br>(25.84 to 108.19)	0<br>(0 to 0)	0<br>(0 to 0)	0.01<br>(-0.01 to 0.04)
Chad	631.51<br>(343.25 to 1073.6)	229.22<br>(109.12 to 436.45)	65.42<br>(30.78 to 123.02)	-0.01<br>(-0.01 to -0.01)	-0.01<br>(-0.01 to -0.01)	0.01<br>(0 to 0.02)

Chile	696.11<br>(382 to 1182.2)	247.81<br>(120.72 to 467.36)	72.31<br>(33.21 to 134.69)	-0.01<br>(-0.01 to -0.01)	-0.01<br>(-0.01 to -0.01)	0<br>(-0.01 to 0.01)
China	362.27<br>(193.47 to 624.76)	143.05<br>(65.83 to 270.82)	38.22<br>(16.96 to 72.01)	-0.01<br>(-0.01 to -0.01)	-0.02<br>(-0.02 to -0.02)	0<br>(-0.01 to 0)
Colombia	661.8<br>(355.29 to 1121.61)	255.09<br>(120.47 to 477.76)	69.13<br>(30.97 to 128.39)	-0.01<br>(-0.01 to -0.01)	-0.01<br>(-0.01 to -0.01)	-0.01<br>(-0.02 to 0)
Comoros	409.36<br>(220.26 to 687.16)	146.33<br>(70.36 to 269.29)	42.81<br>(18.8 to 79.89)	0<br>(0 to 0)	0<br>(0 to 0)	0.02<br>(0.01 to 0.03)
Congo	563.68<br>(301.48 to 961.09)	206.71<br>(99.27 to 399.32)	58.49<br>(25.14 to 110.68)	0<br>(0 to 0)	0<br>(0 to 0)	0.01<br>(0 to 0.01)
Cook Islands	372.01<br>(198.32 to 638.14)	146.6<br>(68.65 to 282.58)	38.97<br>(17.21 to 72.28)	0.01<br>(0.01 to 0.01)	0.01<br>(0.01 to 0.02)	0.01<br>(-0.01 to 0.03)

Costa Rica	663.1<br>(355.92 to 1122.83)	255.54<br>(120.68 to 478.43)	69.35<br>(30.12 to 130.04)	0<br>(0 to 0)	0<br>(0 to 0)	0<br>(-0.01 to 0.01)
Coted'Ivoire	624.3<br>(339.23 to 1059.63)	226.77<br>(108.08 to 432.66)	64.79<br>(29.54 to 120.11)	-0.03<br>(-0.03 to -0.03)	-0.03<br>(-0.03 to -0.03)	-0.01<br>(-0.02 to 0.01)
Croatia	644.62<br>(344.34 to 1088.78)	244.97<br>(117.24 to 456.04)	67.45<br>(30.97 to 126.17)	0<br>(0 to 0)	0<br>(0 to 0)	0.01<br>(0 to 0.02)
Cuba	661.46<br>(355.11 to 1121.2)	254.96<br>(120.41 to 477.54)	69.17<br>(30.9 to 127.03)	0<br>(0 to 0)	0<br>(0 to 0)	0<br>(0 to 0.01)
Cyprus	912.05<br>(516.4 to 1522.33)	290.51<br>(146.29 to 540.59)	95.42<br>(45.37 to 169.97)	0<br>(0 to 0)	0<br>(0 to 0)	0<br>(-0.02 to 0.02)
Czechia	644.72<br>(344.39 to 1088.95)	245.01<br>(117.26 to 456.11)	67.36<br>(30.19 to 128.97)	0<br>(0 to 0)	0<br>(0 to 0)	0.01<br>(0 to 0.02)

Democratic People's Republic of Korea	337.12<br>(178.96 to 582.57)	130.91<br>(60.98 to 261.81)	35.55<br>(15.43 to 70.33)	-0.01<br>(-0.01 to -0.01)	-0.02<br>(-0.02 to -0.02)	0<br>(-0.01 to 0.01)
Democratic Republic of the Congo	561.79<br>(300.42 to 958.68)	206.04<br>(98.93 to 397.97)	58.13<br>(25.33 to 108.01)	0<br>(0 to 0)	0<br>(0 to 0)	0.02<br>(-0.01 to 0.05)
Denmark	1107.56<br>(624.67 to 1847.68)	344.19<br>(172.05 to 647.98)	115.31<br>(55.63 to 205.12)	-0.01<br>(-0.67 to 0.65)	-0.01<br>(-0.51 to 0.48)	-0.02<br>(-0.66 to 0.63)
Djibouti	405.83<br>(218.7 to 679.75)	144.9<br>(69.68 to 265.82)	42.33<br>(19.12 to 77.86)	-0.02<br>(-0.02 to -0.01)	-0.02<br>(-0.02 to -0.01)	-0.02<br>(-0.03 to -0.01)
Dominica	661.82<br>(355.31 to 1121.72)	255.11<br>(120.47 to 477.8)	68.89<br>(31.36 to 127.09)	0<br>(0 to 0)	0<br>(0 to 0)	0.01<br>(0 to 0.02)
Dominican Republic	662.81<br>(355.78 to 1122.55)	255.44<br>(120.63 to 478.28)	69<br>(30.13 to 126.78)	-0.01<br>(-0.01 to -0.01)	-0.01<br>(-0.01 to -0.01)	0<br>(-0.01 to 0.01)

Ecuador	662.61<br>(355.69 to 1122.39)	255.38<br>(120.6 to 478.18)	69.13<br>(31 to 128.98)	-0.01<br>(-0.01 to 0)	-0.01<br>(-0.01 to 0)	0<br>(-0.01 to 0.01)
Egypt	607<br>(332.25 to 1009.35)	222.41<br>(107.41 to 410.85)	63.09<br>(28.98 to 118.63)	0<br>(-0.01 to 0)	-0.01<br>(-0.01 to 0)	0<br>(-0.08 to 0.08)
El Salvador	662.46<br>(355.58 to 1122.08)	255.3<br>(120.57 to 478.06)	69.31<br>(30.73 to 129.93)	-0.01<br>(-0.01 to -0.01)	-0.01<br>(-0.01 to -0.01)	0.01<br>(-0.01 to 0.03)
Equatorial Guinea	548.46<br>(293.26 to 937.1)	201.16<br>(96.45 to 388.25)	57.06<br>(25.02 to 107.29)	-0.1<br>(-0.1 to -0.09)	-0.09<br>(-0.1 to -0.09)	-0.07<br>(-0.08 to -0.06)
Eritrea	408.44<br>(219.81 to 685.2)	145.96<br>(70.18 to 268.39)	42.6<br>(19.45 to 80.96)	0<br>(0 to 0)	0<br>(0 to 0)	0.03<br>(0.02 to 0.05)
Estonia	644.8<br>(344.43 to 1089.07)	245.03<br>(117.27 to 456.17)	67.81<br>(30.83 to 125.85)	0<br>(0 to 0)	0<br>(0 to 0)	0.01<br>(-0.01 to 0.02)

Eswatini	560.74<br>(299.91 to 957.19)	205.61<br>(98.68 to 397.14)	58.44<br>(26.52 to 110.68)	-0.03<br>(-0.03 to -0.03)	-0.03<br>(-0.03 to -0.02)	-0.03<br>(-0.05 to -0.01)
Ethiopia	479.26<br>(255.93 to 829.77)	176.55<br>(82.66 to 335.82)	50<br>(23.27 to 94.13)	0<br>(-0.01 to 0.01)	0<br>(-0.01 to 0.01)	0.02<br>(-0.05 to 0.09)
Fiji	371.11<br>(197.87 to 635.44)	146.11<br>(68.47 to 281.62)	38.93<br>(16.76 to 74.08)	0<br>(0 to 0)	0<br>(0 to 0)	0.01<br>(-0.06 to 0.08)
Finland	777.8<br>(437.23 to 1300.53)	251.72<br>(127.41 to 463.9)	81.31<br>(37.35 to 147.42)	0.83<br>(0.56 to 1.09)	0.64<br>(0.42 to 0.85)	0.83<br>(0.6 to 1.07)
France	711.82<br>(399.04 to 1193.36)	233.69<br>(118.59 to 435.81)	74<br>(34.01 to 132.03)	0<br>(0 to 0)	0<br>(0 to 0)	-0.01<br>(-0.07 to 0.04)
Gabon	567.92<br>(303.84 to 967.21)	208.25<br>(100.01 to 402.15)	58.92<br>(25.88 to 111.54)	0.01<br>(0.01 to 0.02)	0.01<br>(0.01 to 0.02)	0.03<br>(0.01 to 0.05)

Gambia	632.4<br>(343.75 to 1075.1)	229.51<br>(109.25 to 436.91)	65.54<br>(29.95 to 124.47)	0<br>(0 to 0)	0<br>(0 to 0)	0<br>(-0.01 to 0.02)
Georgia	1775.82<br>(965.25 to 3005.81)	643.72<br>(310.65 to 1190.36)	184.45<br>(88.03 to 345.25)	2.71<br>(2.55 to 2.87)	2.6<br>(2.45 to 2.74)	2.69<br>(2.55 to 2.84)
Germany	911.61<br>(516.25 to 1521.31)	290.66<br>(146.39 to 540.82)	94.75<br>(45.28 to 171.97)	-0.01<br>(-0.01 to -0.01)	0<br>(-0.01 to 0)	-0.01<br>(-0.03 to 0)
Ghana	628.44<br>(341.54 to 1068.11)	228.17<br>(108.68 to 434.83)	65.54<br>(29.48 to 123.57)	0<br>(0 to 0)	0<br>(0 to 0)	0.03<br>(0.02 to 0.04)
Greece	1102<br>(623.1 to 1843.79)	342.06<br>(169.22 to 640.83)	114.87<br>(54.62 to 204.36)	0<br>(-0.01 to 0.01)	0<br>(-0.01 to 0.01)	-0.01<br>(-0.05 to 0.04)
Greenland	696.79<br>(385.79 to 1187.77)	248.2<br>(119.49 to 468.18)	72.56<br>(32.91 to 132.6)	0.02<br>(0.01 to 0.02)	0.01<br>(0.01 to 0.02)	0.03<br>(0.01 to 0.04)

Grenada	660.4<br>(354.6 to 1120.05)	254.61<br>(120.24 to 477.04)	68.97<br>(30.83 to 126.38)	-0.01<br>(-0.01 to -0.01)	-0.01<br>(-0.01 to -0.01)	-0.01<br>(-0.02 to 0)
Guam	370.95<br>(197.79 to 634.95)	146.02<br>(68.44 to 281.44)	38.8<br>(15.99 to 73.39)	0<br>(0 to 0.01)	0.01<br>(0 to 0.01)	0.01<br>(0 to 0.03)
Guatemala	663.5<br>(356.12 to 1123.25)	255.69<br>(120.75 to 478.74)	68.93<br>(30.67 to 125.68)	-0.01<br>(-0.01 to -0.01)	-0.01<br>(-0.01 to -0.01)	0.01<br>(0 to 0.02)
Guinea	632.23<br>(343.67 to 1074.8)	229.38<br>(109.16 to 436.63)	65.62<br>(29.7 to 120.99)	0.01<br>(0 to 0.01)	0.01<br>(0 to 0.01)	0.01<br>(-0.02 to 0.04)
Guinea-Bissau	630.23<br>(342.54 to 1071.4)	228.78<br>(108.94 to 435.78)	65.5<br>(29.13 to 122.72)	-0.01<br>(-0.01 to -0.01)	-0.01<br>(-0.01 to -0.01)	0.01<br>(0 to 0.03)
Guyana	662.86<br>(355.81 to 1122.65)	255.47<br>(120.64 to 478.33)	68.52<br>(29.85 to 127.12)	-0.01<br>(-0.01 to -0.01)	-0.01<br>(-0.01 to -0.01)	0<br>(-0.01 to 0.01)

Haiti	663.32<br>(356.04 to 1123.14)	255.64<br>(120.72 to 478.58)	68.5<br>(30.03 to 127.63)	-0.01<br>(-0.01 to -0.01)	-0.01<br>(-0.01 to -0.01)	0<br>(-0.02 to 0.03)
Honduras	663.04<br>(355.88 to 1122.73)	255.52<br>(120.67 to 478.4)	69.23<br>(30.39 to 129.37)	0<br>(0 to 0)	0<br>(0 to 0)	0<br>(-0.03 to 0.03)
Hungary	644.61<br>(344.34 to 1088.77)	244.97<br>(117.24 to 456.03)	67.71<br>(29.9 to 126.75)	0<br>(0 to 0)	0<br>(0 to 0)	0.01<br>(0 to 0.02)
Iceland	915.12<br>(518.24 to 1527.92)	291.22<br>(146.56 to 542.11)	95.42<br>(44.63 to 173.32)	0<br>(-0.01 to 0.01)	0<br>(0 to 0)	0<br>(0 to 0.01)
India	374<br>(200.3 to 644.12)	136.1<br>(65.28 to 255.86)	38.87<br>(18.15 to 71.82)	0<br>(-0.09 to 0.09)	0<br>(-0.13 to 0.12)	0.02<br>(-0.07 to 0.1)
Indonesia	404.13<br>(214.03 to 688.19)	160.21<br>(75.39 to 314.39)	42.44<br>(19.32 to 80.4)	-0.01<br>(-0.01 to -0.01)	-0.01<br>(-0.01 to -0.01)	0<br>(0 to 0.01)

Iran (Islamic Republic of)	754.02<br>(406.98 to 1258.88)	285.23<br>(137.13 to 551.84)	78.47<br>(37.07 to 148.21)	-0.01<br>(-0.04 to 0.02)	-0.01<br>(-0.03 to 0.02)	0<br>(-0.04 to 0.03)
Iraq	609.07<br>(333.39 to 1012.99)	223.25<br>(107.77 to 412.63)	63.67<br>(28.45 to 116.5)	0<br>(-0.01 to 0.02)	0<br>(-0.01 to 0.02)	0.02<br>(-0.06 to 0.1)
Ireland	915.01<br>(518.27 to 1527.48)	291.26<br>(146.62 to 542.09)	95.15<br>(45.25 to 168.61)	0<br>(0 to 0)	0<br>(0 to 0)	0<br>(-0.01 to 0.01)
Israel	914.37<br>(517.87 to 1526.35)	291.11<br>(146.55 to 541.78)	95.59<br>(45.93 to 172.15)	0<br>(0 to 0)	0<br>(0 to 0)	0.01<br>(0.01 to 0.02)
Italy	1024.05<br>(569.07 to 1761.55)	350.62<br>(173.42 to 650.05)	106.59<br>(50.14 to 195.44)	-0.01<br>(-0.01 to -0.01)	0<br>(0 to 0)	-0.01<br>(-0.02 to 0)
Jamaica	662.36<br>(355.57 to 1122.19)	255.3<br>(120.56 to 478.07)	68.93<br>(31.96 to 129.65)	-0.01<br>(-0.01 to -0.01)	-0.01<br>(-0.01 to -0.01)	-0.02<br>(-0.03 to -0.01)

Japan	803.44<br>(436.95 to 1378.18)	293.98<br>(143.5 to 555)	84.13<br>(39.76 to 157.55)	0<br>(0 to 0)	0.01<br>(0 to 0.01)	0<br>(-0.01 to 0.02)
Jordan	607.27<br>(332.36 to 1009.75)	222.61<br>(107.48 to 411.34)	63.15<br>(28.59 to 115.75)	0.01<br>(0 to 0.02)	0.01<br>(0 to 0.02)	0.01<br>(-0.03 to 0.04)
Kazakhstan	645.13<br>(344.6 to 1089.59)	245.14<br>(117.33 to 456.41)	67.4<br>(29.96 to 125.62)	0<br>(0 to 0)	0<br>(0 to 0)	0.01<br>(0.01 to 0.02)
Kenya	479.57<br>(256.1 to 830.4)	176.69<br>(82.71 to 336)	50.07<br>(23.23 to 93.44)	-0.01<br>(-0.01 to -0.01)	-0.01<br>(-0.01 to -0.01)	0<br>(-0.01 to 0.01)
Kiribati	371.48<br>(198.05 to 636.58)	146.31<br>(68.55 to 282.02)	38.83<br>(17.23 to 73.59)	0<br>(0 to 0)	0<br>(0 to 0)	0.01<br>(0 to 0.02)
Kuwait	574.22<br>(312.1 to 958.34)	211.68<br>(101.24 to 398.85)	60.25<br>(27.26 to 111.1)	-0.05<br>(-0.07 to -0.03)	-0.05<br>(-0.07 to -0.03)	-0.05<br>(-0.12 to 0.02)

Kyrgyzstan	645.15<br>(344.61 to 1089.62)	245.15<br>(117.33 to 456.44)	67.31<br>(30.93 to 125.29)	0<br>(0 to 0)	0<br>(0 to 0)	0<br>(-0.01 to 0.01)
Lao People's Democratic Republic	342.46<br>(183.86 to 578.6)	132.78<br>(61.13 to 254.95)	35.99<br>(16.2 to 67.31)	-0.01<br>(-0.01 to -0.01)	-0.01<br>(-0.01 to -0.01)	0.03<br>(0.01 to 0.04)
Latvia	644.67<br>(344.37 to 1088.87)	244.99<br>(117.25 to 456.06)	67.52<br>(29.71 to 128.93)	0<br>(0 to 0)	0<br>(0 to 0)	0<br>(-0.01 to 0.01)
Lebanon	604.94<br>(331.12 to 1005.69)	221.56<br>(107.06 to 409.07)	62.82<br>(28.18 to 115.32)	-0.02<br>(-0.03 to 0)	-0.02<br>(-0.03 to 0)	-0.01<br>(-0.07 to 0.04)
Lesotho	562.47<br>(300.8 to 959.57)	206.3<br>(99.06 to 398.47)	58.39<br>(25.12 to 109.26)	-0.03<br>(-0.03 to -0.03)	-0.03<br>(-0.03 to -0.02)	-0.03<br>(-0.04 to -0.02)
Liberia	629.09<br>(341.9 to 1069.34)	228.39<br>(108.77 to 435.18)	64.79<br>(29.51 to 121.2)	-0.04<br>(-0.06 to -0.02)	-0.04<br>(-0.05 to -0.02)	-0.02<br>(-0.03 to 0)

Libya	610.89<br>(334.4 to 1015.76)	223.96<br>(108.07 to 414.09)	63.48<br>(28.55 to 118.04)	-0.02<br>(-0.03 to -0.01)	-0.02<br>(-0.04 to -0.01)	-0.02<br>(-0.05 to 0.01)
Lithuania	644.8<br>(344.44 to 1089.08)	245.04<br>(117.28 to 456.18)	67.45<br>(30.42 to 127.87)	0<br>(0 to 0)	0<br>(0 to 0)	-0.01<br>(-0.06 to 0.04)
Luxembourg	912.35<br>(516.61 to 1522.83)	290.63<br>(146.35 to 540.82)	94.96<br>(45.11 to 165.97)	-0.01<br>(-0.01 to 0)	-0.01<br>(-0.01 to 0)	-0.01<br>(-0.02 to 0)
Madagascar	410.12<br>(220.62 to 688.92)	146.63<br>(70.51 to 270.05)	42.76<br>(19.36 to 80.15)	0<br>(0 to 0)	0<br>(0 to 0)	0.03<br>(0.02 to 0.04)
Malawi	410.18<br>(220.64 to 689.08)	146.67<br>(70.52 to 270.13)	42.52<br>(18.9 to 78.45)	0<br>(0 to 0)	0<br>(0 to 0)	0.05<br>(0.03 to 0.06)
Malaysia	342.17<br>(183.73 to 578.22)	132.64<br>(61.07 to 254.67)	35.82<br>(15.17 to 68.78)	0.16<br>(0.16 to 0.17)	0.19<br>(0.19 to 0.2)	0.16<br>(0.07 to 0.25)

Maldives	341.45	132.3	35.74	-0.01	-0.01	0.01
	(183.35 to 577.29)	(60.9 to 254.11)	(15.86 to 67.02)	(-0.01 to -0.01)	(-0.02 to -0.01)	(-0.06 to 0.08)
Mali	629.72	228.61	65.2	0	0	0.01
	(342.26 to 1070.49)	(108.86 to 435.51)	(29.7 to 121)	(-0.01 to 0)	(-0.01 to 0)	(-0.01 to 0.02)
Malta	913.16	290.64	95.41	0	0	0
	(517.04 to 1524.38)	(146.33 to 540.87)	(45.17 to 170.34)	(-0.01 to 0.01)	(0 to 0)	(-0.02 to 0.01)
Marshall Islands	371.23	146.18	39.05	0	0	0
	(197.92 to 635.85)	(68.5 to 281.76)	(17.1 to 73.46)	(0 to 0)	(0 to 0)	(-0.02 to 0.01)
Mauritania	632.33	229.46	65.8	0.01	0.01	0.02
	(343.71 to 1074.97)	(109.21 to 436.79)	(29.36 to 122.32)	(0 to 0.01)	(0 to 0.01)	(0.01 to 0.03)
Mauritius	342.48	132.8	35.75	0	0	0
	(183.86 to 578.64)	(61.14 to 254.99)	(15.69 to 65.48)	(0 to 0)	(0 to 0)	(-0.01 to 0.01)

Mexico	772.71<br>(416.53 to 1307.08)	306.69<br>(143.88 to 593.93)	80.9<br>(37.5 to 151.04)	-0.01<br>(-0.01 to -0.01)	0<br>(-0.01 to 0)	0<br>(-0.01 to 0.01)
Micronesia (Federated States of)	370.99<br>(197.83 to 635.05)	146.04<br>(68.45 to 281.47)	38.85<br>(17.43 to 73.13)	0<br>(0 to 0)	0<br>(0 to 0)	-0.01<br>(-0.09 to 0.07)
Monaco	915.1<br>(518.36 to 1527.65)	291.57<br>(146.75 to 542.71)	95.18<br>(46.11 to 172.62)	0.01<br>(-0.01 to 0.03)	0.02<br>(0.01 to 0.02)	0.01<br>(-0.02 to 0.04)
Mongolia	645.1<br>(344.58 to 1089.55)	245.13<br>(117.33 to 456.41)	67.74<br>(30.67 to 129.52)	0<br>(0 to 0)	0<br>(0 to 0)	0.02<br>(0.01 to 0.03)
Montenegro	643.89<br>(343.97 to 1087.4)	244.71<br>(117.11 to 455.56)	67.37<br>(29.97 to 126.51)	0<br>(-0.01 to 0)	0<br>(0 to 0)	-0.01<br>(-0.01 to 0)
Morocco	610.95<br>(334.45 to 1015.83)	223.97<br>(108.07 to 414.1)	63.65<br>(29.17 to 117.48)	-0.02<br>(-0.02 to -0.02)	-0.03<br>(-0.03 to -0.02)	-0.03<br>(-0.04 to -0.02)

Mozambique	410.83	146.9	42.47	0	0	0.01
	(221.01 to 690.63)	(70.64 to 270.74)	(18.82 to 79.64)	(0 to 0)	(-0.01 to 0)	(0 to 0.03)
Myanmar	342.63	132.87	35.69	0	0	0.01
	(183.94 to 578.83)	(61.17 to 255.12)	(16.3 to 67.92)	(0 to 0)	(0 to 0)	(0 to 0.02)
Namibia	563.78	206.77	58.67	0	0	0
	(301.55 to 961.26)	(99.31 to 399.42)	(26.91 to 110.31)	(0 to 0)	(0 to 0)	(-0.01 to 0.01)
Nauru	370.99	146.03	38.77	0	0	-0.01
	(197.84 to 635)	(68.45 to 281.45)	(17 to 74.47)	(0 to 0)	(0 to 0)	(-0.01 to 0)
Nepal	323.92	114.32	33.88	0	0	0.03
	(173.49 to 551.12)	(55.82 to 217.66)	(15.03 to 63.97)	(0 to 0)	(0 to 0)	(0.02 to 0.04)
Netherlands	814.55	263.29	84.93	0	0	-0.01
	(459.79 to 1353.77)	(131.9 to 485.43)	(39.15 to 149.93)	(-0.01 to 0.01)	(-0.01 to 0.01)	(-0.06 to 0.04)
New Zealand	343.11	123.06	35.77	0	0	0.01
	(173.43 to 621.52)	(56.89 to 246.49)		(0 to 0.01)	(0 to 0.01)	

			(15.05 to 70.94)			(-0.1 to 0.12)
Nicaragua	661.97<br>(355.35 to 1121.69)	255.14<br>(120.49 to 477.83)	69.27<br>(29.62 to 128.83)	-0.01<br>(-0.01 to -0.01)	-0.01<br>(-0.01 to -0.01)	0.01<br>(0 to 0.02)
Niger	632.2<br>(343.65 to 1074.76)	229.39<br>(109.17 to 436.64)	65.71<br>(29.65 to 123.54)	-0.02<br>(-0.02 to -0.01)	-0.02<br>(-0.02 to -0.02)	0<br>(-0.01 to 0.01)
Nigeria	731.05<br>(398.95 to 1234.68)	275.89<br>(132.09 to 511.87)	75.75<br>(35.87 to 141.32)	-0.01<br>(-0.02 to -0.01)	-0.02<br>(-0.02 to -0.01)	0.01<br>(-0.01 to 0.03)
Niue	369.91<br>(197.36 to 632.68)	145.41<br>(68.22 to 280.33)	38.91<br>(17.58 to 73.26)	0<br>(0 to 0)	0<br>(0 to 0)	0<br>(-0.01 to 0.01)
North Macedonia	644.34<br>(344.2 to 1088.32)	244.87<br>(117.19 to 455.84)	67.45<br>(30.24 to 126.06)	0<br>(0 to 0)	0<br>(0 to 0)	0.01<br>(-0.01 to 0.02)
Northern Mariana Islands	370.93<br>(197.82 to 634.78)	146<br>(68.43 to 281.36)	38.79<br>(17.22 to 74.46)	-0.02<br>(-0.04 to 0)	-0.02<br>(-0.05 to 0)	-0.03<br>(-0.07 to 0.01)

Norway	805.89	280.04	83.92	0.38	0.44	0.38
	(444.9 to 1334.39)	(138.8 to 509.28)	(39.7 to 150.33)	(0.33 to 0.43)	(0.38 to 0.5)	(0.33 to 0.42)
Oman	611.46	224.21	63.6	0.05	0.05	0.04
	(334.71 to 1016.64)	(108.17 to 414.61)	(29.2 to 116.84)	(0.05 to 0.06)	(0.05 to 0.06)	(0.01 to 0.07)
Pakistan	373.84	135.99	38.89	0	0	0.01
	(201.36 to 642.11)	(65.35 to 254.6)	(17.4 to 72.26)	(0 to 0)	(0 to 0)	(0 to 0.01)
Palau	370.81	145.95	38.96	0	0	0
	(197.72 to 634.59)	(68.42 to 281.32)	(17.21 to 74.31)	(0 to 0)	(-0.01 to 0)	(-0.01 to 0.01)
Palestine	610.5	223.81	63.67	0	0	0.02
	(334.19 to 1015.19)	(108 to 413.79)	(29.04 to 116.12)	(0 to 0)	(0 to 0)	(0.01 to 0.03)
Panama	661.92	255.13	69.28	0	0	0
	(355.34 to 1121.68)	(120.48 to 477.8)	(31.09 to 127.53)	(0 to 0)	(0 to 0)	(-0.01 to 0.01)
Papua New Guinea	370.61	145.81	38.85	0	0	0.02
	(197.68 to 633.88)	(68.36 to 280.98)		(0 to 0)	(0 to 0)	

			(17.13 to 75.13)			(0.01 to 0.03)
Paraguay	642.62 (349.47 to 1100.3)	248.68 (116.1 to 485.18)	66.86 (30.36 to 126.59)	-0.01 (-0.01 to -0.01)	-0.01 (-0.01 to -0.01)	0 (-0.01 to 0.01)
Peru	659.99 (354.36 to 1119.43)	254.44 (120.16 to 476.79)	68.69 (30.93 to 128.47)	-0.02 (-0.02 to -0.02)	-0.02 (-0.02 to -0.02)	-0.01 (-0.02 to 0)
Philippines	404.27 (214.13 to 688.52)	160.27 (75.43 to 314.5)	42.28 (19.29 to 80.53)	0 (0 to 0)	-0.01 (-0.01 to 0)	0 (-0.01 to 0.01)
Poland	741.14 (403.05 to 1245.54)	289.47 (137.22 to 539.02)	77.67 (36.23 to 146.19)	0 (0 to 0)	0 (0 to 0)	0 (0 to 0.01)
Portugal	914.73 (518.1 to 1527)	291.21 (146.59 to 541.99)	95.29 (43.61 to 168.96)	0 (-0.01 to 0)	0 (0 to 0)	0 (-0.01 to 0)
Puerto Rico	662.22 (355.49 to 1121.99)	255.24 (120.53 to 477.96)	69.18 (30.33 to 127.51)	0 (0 to 0)	0 (0 to 0)	0 (-0.02 to 0.02)

Qatar	611.06<br>(334.5 to 1016.03)	224.03<br>(108.09 to 414.21)	63.7<br>(29.1 to 116.73)	0.08<br>(0.04 to 0.12)	0.08<br>(0.02 to 0.14)	0.07<br>(0.02 to 0.12)
Republic of Korea	621.75<br>(341.83 to 1038.76)	218.66<br>(108.1 to 404.3)	65.15<br>(29.84 to 115.91)	0<br>(-0.01 to 0)	0<br>(-0.01 to 0.01)	-0.01<br>(-0.04 to 0.02)
Republic of Moldova	644.54<br>(344.31 to 1088.66)	244.95<br>(117.23 to 455.98)	67.67<br>(30.4 to 127.44)	0<br>(0 to 0)	0<br>(0 to 0)	0<br>(-0.01 to 0.01)
Romania	644.58<br>(344.32 to 1088.72)	244.96<br>(117.24 to 456.01)	67.35<br>(31.05 to 126.04)	0<br>(0 to 0)	0<br>(0 to 0)	0<br>(-0.06 to 0.06)
Russian Federation	741.18<br>(403.07 to 1245.61)	289.48<br>(137.23 to 539.05)	77.56<br>(36.02 to 144.82)	0<br>(0 to 0)	0<br>(0 to 0)	0<br>(0 to 0.01)
Rwanda	409.96<br>(220.55 to 688.53)	146.57<br>(70.48 to 269.89)	42.86<br>(18.75 to 78.78)	0<br>(-0.01 to 0)	0<br>(-0.01 to 0)	0.03<br>(-0.01 to 0.06)

Saint Kitts and Nevis	663.3<br>(356.03 to 1123.12)	255.63<br>(120.72 to 478.57)	69.06<br>(29.54 to 128.73)	0<br>(0 to 0)	0<br>(0 to 0)	0.01<br>(0 to 0.02)
Saint Lucia	661.62<br>(355.2 to 1121.46)	255.03<br>(120.44 to 477.66)	68.71<br>(30.07 to 126.56)	-0.01<br>(-0.01 to -0.01)	-0.01<br>(-0.01 to -0.01)	-0.01<br>(-0.02 to 0)
Saint Vincent and the Grenadines	661.89<br>(355.34 to 1121.78)	255.14<br>(120.48 to 477.84)	69.03<br>(30.36 to 128.83)	0<br>(0 to 0)	0<br>(0 to 0)	0<br>(0 to 0.01)
Samoa	370.98<br>(197.82 to 634.99)	146.03<br>(68.44 to 281.45)	38.74<br>(17.49 to 72.62)	0.01<br>(0.01 to 0.01)	0.01<br>(0.01 to 0.01)	0<br>(-0.05 to 0.05)
San Marino	912.06<br>(516.39 to 1522.37)	290.43<br>(146.25 to 540.4)	94.94<br>(45.47 to 170.7)	-0.02<br>(-0.02 to -0.02)	-0.01<br>(-0.01 to -0.01)	-0.02<br>(-0.03 to -0.02)
Sao Tome and Principe	630.72<br>(342.8 to 1072.24)	228.99<br>(109.04 to 436.15)	65.67<br>(30.03 to 122.28)	0.01<br>(0.01 to 0.01)	0.01<br>(0.01 to 0.01)	0.01<br>(0 to 0.03)

Saudi Arabia	595.85<br>(325.98 to 989.45)	218.16<br>(105.51 to 402.33)	62.21<br>(28.33 to 115.22)	-0.04<br>(-0.04 to -0.03)	-0.04<br>(-0.05 to -0.04)	-0.06<br>(-0.07 to -0.04)
Senegal	623.54<br>(338.81 to 1058.01)	226.54<br>(107.99 to 432.34)	64.63<br>(29.35 to 119.46)	-0.06<br>(-0.06 to -0.06)	-0.06<br>(-0.06 to -0.06)	-0.05<br>(-0.06 to -0.04)
Serbia	643.09<br>(343.54 to 1085.43)	244.39<br>(116.92 to 455.11)	67.08<br>(30.62 to 125.7)	-0.01<br>(-0.01 to -0.01)	-0.01<br>(-0.01 to -0.01)	-0.01<br>(-0.02 to 0)
Seychelles	342.27<br>(183.76 to 578.38)	132.7<br>(61.09 to 254.8)	35.94<br>(16.61 to 69.71)	0<br>(0 to 0)	0<br>(-0.01 to 0)	0<br>(-0.02 to 0.02)
Sierra Leone	632.73<br>(343.93 to 1075.66)	229.65<br>(109.31 to 437.15)	65.59<br>(28.78 to 121.4)	0<br>(-0.01 to 0.01)	0.01<br>(0 to 0.01)	0.03<br>(0 to 0.07)
Singapore	658.19<br>(358.43 to 1079.79)	230.93<br>(114.33 to 426.57)	68.9<br>(31.5 to 122.62)	0.01<br>(0 to 0.03)	0.01<br>(0 to 0.03)	0.01<br>(-0.04 to 0.05)

Slovakia	644.67<br>(344.37 to 1088.86)	244.99<br>(117.25 to 456.07)	67.28<br>(30.21 to 126.98)	0<br>(0 to 0)	0<br>(0 to 0)	0<br>(-0.02 to 0.01)
Slovenia	644.5<br>(344.28 to 1088.59)	244.93<br>(117.22 to 455.95)	67.4<br>(30.43 to 125.09)	0<br>(0 to 0)	0<br>(0 to 0)	0<br>(0 to 0.01)
Solomon Islands	370.88<br>(197.8 to 634.62)	145.97<br>(68.42 to 281.3)	38.87<br>(17.25 to 74.42)	0<br>(0 to 0)	0<br>(0 to 0)	0.01<br>(0 to 0.02)
Somalia	408.19<br>(219.68 to 684.66)	145.86<br>(70.13 to 268.14)	42.36<br>(19.01 to 78.77)	0.01<br>(0.01 to 0.01)	0.01<br>(0.01 to 0.01)	0.03<br>(0.02 to 0.04)
South Africa	652.58<br>(354.84 to 1117.78)	247.56<br>(118.72 to 472.69)	67.81<br>(31.98 to 126.7)	-0.02<br>(-0.02 to -0.02)	-0.02<br>(-0.02 to -0.02)	-0.01<br>(-0.02 to -0.01)
South Sudan	408.29<br>(219.76 to 684.87)	145.89<br>(70.15 to 268.23)	42.4<br>(19.12 to 79.02)	0<br>(0 to 0)	0<br>(0 to 0)	0.02<br>(0.01 to 0.03)

Spain	753.37<br>(422.04 to 1262.36)	246.25<br>(123.57 to 460.9)	78.35<br>(37.34 to 142.8)	0.2<br>(0.07 to 0.33)	0.19<br>(0.07 to 0.31)	0.18<br>(0.03 to 0.33)
Sri Lanka	342.57<br>(183.9 to 578.76)	132.84<br>(61.16 to 255.07)	36.1<br>(16.18 to 68.75)	0<br>(0 to 0)	0<br>(0 to 0)	0.02<br>(0.01 to 0.03)
Sudan	608.73<br>(333.22 to 1012.42)	223.09<br>(107.7 to 412.26)	63.29<br>(28.96 to 116.27)	-0.02<br>(-0.03 to -0.01)	-0.02<br>(-0.03 to -0.01)	-0.01<br>(-0.03 to 0.02)
Suriname	661.46<br>(355.09 to 1121.17)	254.96<br>(120.41 to 477.57)	68.93<br>(31.76 to 127.91)	0<br>(0 to 0)	0<br>(0 to 0)	0<br>(0 to 0.01)
Sweden	926.01<br>(515.71 to 1535.38)	303.76<br>(151.29 to 569.05)	96.33<br>(45.34 to 171.76)	-0.01<br>(-0.03 to 0)	-0.03<br>(-0.05 to -0.01)	-0.01<br>(-0.07 to 0.04)
Switzerland	913.1<br>(517.13 to 1524.01)	290.9<br>(146.48 to 541.32)	94.95<br>(44.89 to 169.69)	0<br>(0 to 0)	0<br>(0 to 0)	0<br>(-0.02 to 0.01)

Syrian Arab Republic	615.18<br>(336.81 to 1022.06)	225.6<br>(108.78 to 417.46)	63.81<br>(28.8 to 117.61)	0.02<br>(0 to 0.03)	0.02<br>(0 to 0.03)	0.02<br>(-0.02 to 0.06)
Taiwan (Province of China)	328.44<br>(263.61 to 411.22)	127.95<br>(86.76 to 174.91)	34.51<br>(20.64 to 52.19)	-0.45<br>(-0.46 to -0.45)	-0.41<br>(-0.42 to -0.41)	-0.45<br>(-0.5 to -0.41)
Tajikistan	644.48<br>(344.27 to 1088.54)	244.92<br>(117.21 to 455.95)	67.3<br>(30.16 to 125.08)	-0.01<br>(-0.01 to -0.01)	-0.01<br>(-0.01 to -0.01)	0.01<br>(0 to 0.02)
Thailand	342.44<br>(183.86 to 578.57)	132.77<br>(61.13 to 254.92)	35.94<br>(15.6 to 68.11)	0<br>(0 to 0)	0<br>(0 to 0)	0<br>(-0.03 to 0.03)
Timor-Leste	342.18<br>(183.74 to 578.22)	132.64<br>(61.07 to 254.66)	35.83<br>(15.68 to 68.27)	0.01<br>(0.01 to 0.01)	0.01<br>(0.01 to 0.01)	0.04<br>(0.02 to 0.05)
Togo	625.16<br>(339.71 to 1061.33)	227.1<br>(108.24 to 433.22)	64.52<br>(28.39 to 120.84)	-0.02<br>(-0.03 to -0.02)	-0.02<br>(-0.02 to -0.02)	0<br>(-0.03 to 0.03)

Tokelau	370.5<br>(197.64 to 633.66)	145.75<br>(68.34 to 280.84)	38.94<br>(16.88 to 75.59)	0<br>(-0.01 to 0.01)	0<br>(-0.02 to 0.02)	0.01<br>(-0.08 to 0.09)
Tonga	370.99<br>(197.85 to 634.94)	146.03<br>(68.44 to 281.42)	38.94<br>(16.95 to 73.43)	0<br>(0 to 0)	0<br>(0 to 0)	0<br>(-0.05 to 0.05)
Trinidad and Tobago	662.39<br>(355.57 to 1122.18)	255.3<br>(120.56 to 478.07)	68.85<br>(31.74 to 127.48)	0<br>(0 to 0)	0<br>(0 to 0)	0.01<br>(-0.05 to 0.07)
Tunisia	608.47<br>(333.1 to 1011.97)	222.93<br>(107.64 to 411.88)	63.1<br>(28.73 to 117.28)	-0.01<br>(-0.02 to -0.01)	-0.02<br>(-0.02 to -0.01)	-0.02<br>(-0.07 to 0.03)
Turkey	609.92<br>(333.86 to 1014.33)	223.59<br>(107.91 to 413.32)	63.58<br>(27.94 to 118.77)	-0.01<br>(-0.01 to 0)	-0.01<br>(-0.01 to 0)	0.01<br>(0 to 0.02)
Turkmenistan	643.88<br>(343.98 to 1087.62)	244.74<br>(117.12 to 455.58)	67.4<br>(31.64 to 125.21)	-0.01<br>(-0.01 to -0.01)	-0.01<br>(-0.01 to 0)	0<br>(-0.01 to 0.01)

Tuvalu	370.47 (197.62 to 633.65)	145.73 (68.34 to 280.83)	38.81 (17.52 to 75.22)	-0.01 (-0.01 to -0.01)	-0.01 (-0.01 to -0.01)	-0.02 (-0.05 to 0.02)
Uganda	409.28 (220.25 to 686.94)	146.28 (70.34 to 269.2)	42.6 (18.53 to 79.11)	-0.01 (-0.01 to -0.01)	-0.01 (-0.01 to -0.01)	0.02 (-0.01 to 0.04)
Ukraine	740.9 (402.96 to 1245.13)	289.37 (137.16 to 538.79)	77.74 (36.36 to 147.1)	0 (0 to 0)	0 (0 to 0)	0.01 (0 to 0.02)
United Arab Emirates	609.58 (333.66 to 1013.87)	223.48 (107.86 to 413.11)	63.28 (28.68 to 117.94)	0.05 (0.01 to 0.08)	0.05 (0.01 to 0.08)	0.06 (-0.03 to 0.15)
United Kingdom	1091.02 (610.54 to 1806.98)	367.2 (183.26 to 678.66)	113.5 (54.43 to 203.93)	0.65 (0.59 to 0.7)	0.73 (0.69 to 0.78)	0.65 (0.59 to 0.71)
United Republic of Tanzania	410.31 (220.74 to 689.36)	146.7 (70.54 to 270.23)	42.57 (18.63 to 77.63)	0 (0 to 0)	0 (0 to 0)	0.05 (0.04 to 0.06)

United States of America	799.48<br>(435.05 to 1356.91)	296.89<br>(143.69 to 550.98)	82.84<br>(39.05 to 151.44)	0<br>(-0.06 to 0.06)	-0.01<br>(-0.06 to 0.04)	-0.02<br>(-0.09 to 0.05)
United States Virgin Islands	662.69<br>(355.71 to 1122.14)	255.36<br>(120.59 to 478.04)	69.12<br>(31.3 to 130.13)	0<br>(0 to 0)	0<br>(0 to 0)	0<br>(-0.05 to 0.06)
Uruguay	696.6<br>(382.31 to 1182.84)	247.9<br>(120.79 to 467.53)	72.8<br>(34.02 to 136.14)	0<br>(0 to 0)	0<br>(0 to 0)	0<br>(-0.01 to 0.01)
Uzbekistan	644.66<br>(344.36 to 1088.83)	244.97<br>(117.24 to 456.07)	67.22<br>(31.08 to 125.19)	-0.01<br>(-0.01 to -0.01)	-0.01<br>(-0.01 to -0.01)	0.01<br>(0 to 0.01)
Vanuatu	371.03<br>(197.87 to 635.1)	146.05<br>(68.45 to 281.48)	38.8<br>(17.31 to 73.28)	0<br>(0 to 0)	0<br>(0 to 0)	0.01<br>(0 to 0.02)
Venezuela (Bolivarian Republic of)	663.61<br>(356.14 to 1123.2)	255.71<br>(120.76 to 478.85)	69.33<br>(30.67 to 131.15)	0<br>(0 to 0.01)	0<br>(0 to 0.01)	0<br>(-0.01 to 0)

Viet Nam	342.04<br>(183.68 to 578.02)	132.57<br>(61.04 to 254.53)	36.01<br>(16.18 to 67.35)	-0.01<br>(-0.01 to -0.01)	-0.01<br>(-0.01 to -0.01)	0.01<br>(0 to 0.02)
Yemen	610.99<br>(334.47 to 1015.86)	223.97<br>(108.08 to 414.08)	62.86<br>(28.67 to 117.37)	0.03<br>(0.02 to 0.03)	0.03<br>(0.02 to 0.03)	0.05<br>(0.03 to 0.06)
Zambia	410.26<br>(220.7 to 689.24)	146.68<br>(70.53 to 270.18)	42.73<br>(19.26 to 79.58)	0<br>(0 to 0)	0<br>(0 to 0)	0.03<br>(0.02 to 0.04)
Zimbabwe	564.1<br>(301.71 to 961.64)	206.87<br>(99.34 to 399.63)	58.81<br>(26.07 to 110.65)	0<br>(0 to 0)	0<br>(0 to 0)	0<br>(-0.01 to 0.01)

Note: Estimates are for Children and Adolescents. AAPCs=average annual percent changes. CI=confidence interval. P=P value for the significant test of AAPCs. NP=neck pain. YLDs=years lived with disability. Numbers in parentheses are 95% uncertainty intervals (Age-standardized rate) and 95% confidence interval (AAPCs).

Supplementary Figure 1. The changes in the proportion of prevalence cases among NP among children and adolescents to the overall NP patients from 1990 to 2021

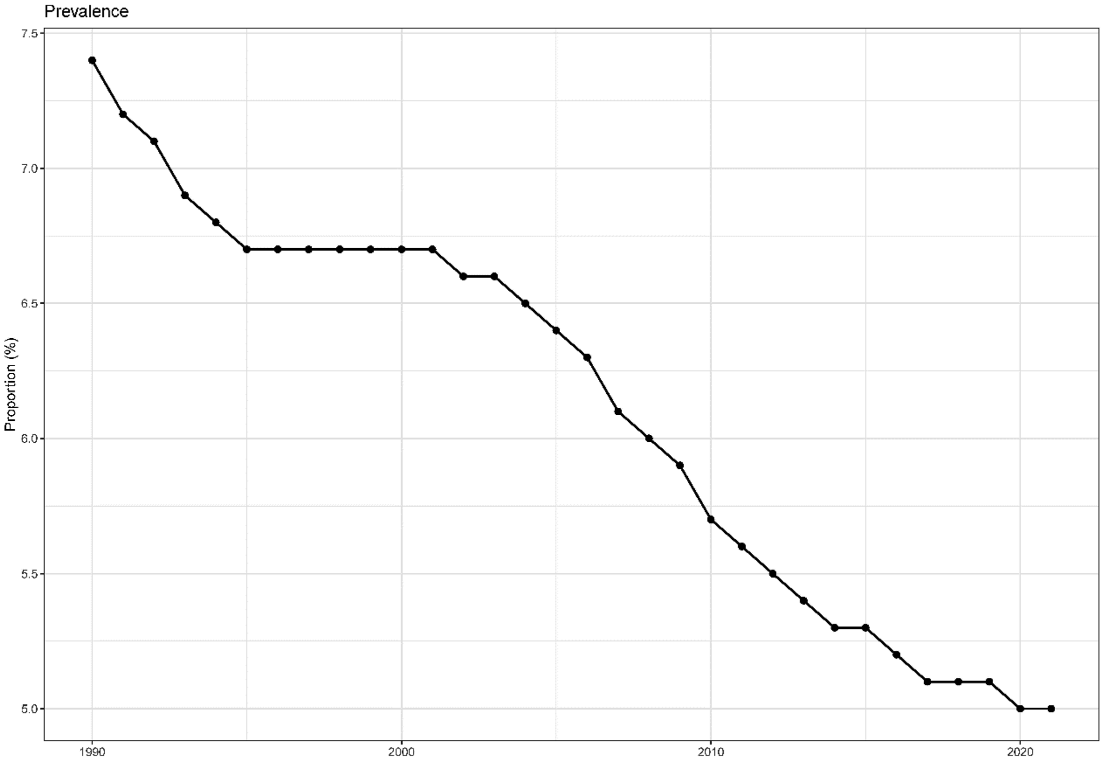

Supplementary Figure 2. Temporal trend of age-standardized Prevalence, incidence and YLDs for children and adolescents with NP, and overall NP patients from 1990 to 2021

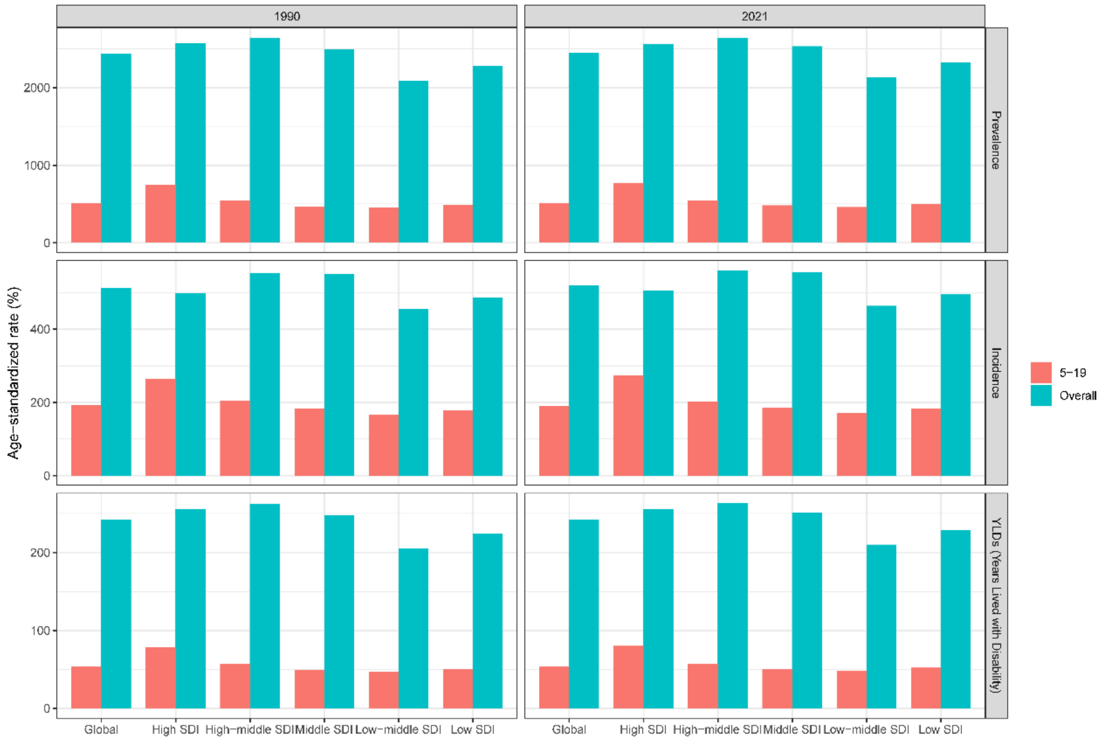

Supplementary Figure 3. The changes in the proportion of incidence (A) and YLDs (B) cases among NP in children and adolescents to the overall NP patients from 1990 to 2021

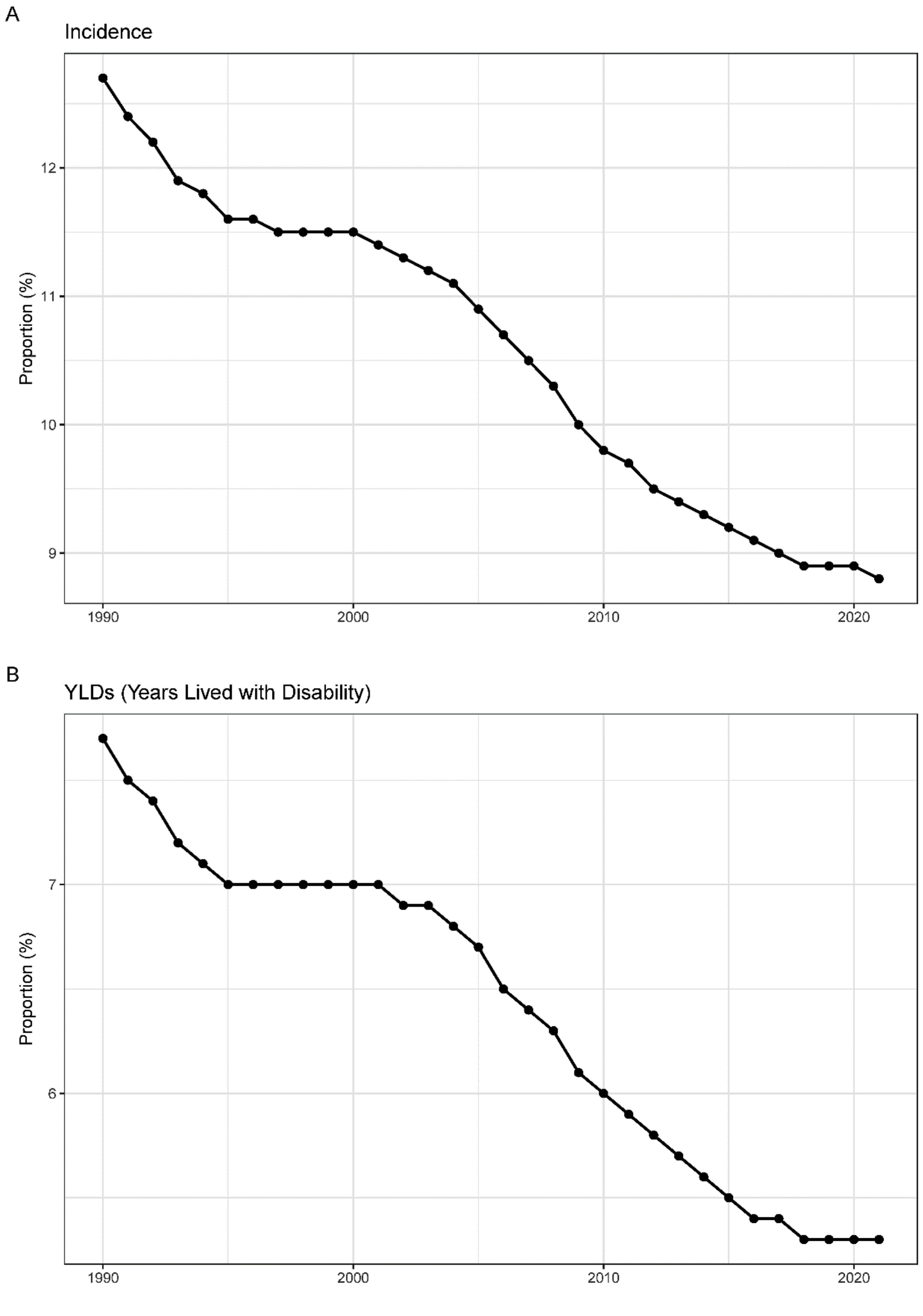

Supplementary Figure 4. Temporal trend of age standardized incidence, prevalence and YLDs of NP among children and adolescents from 1990 to 2021 at global and socio-demographic index levels by sex

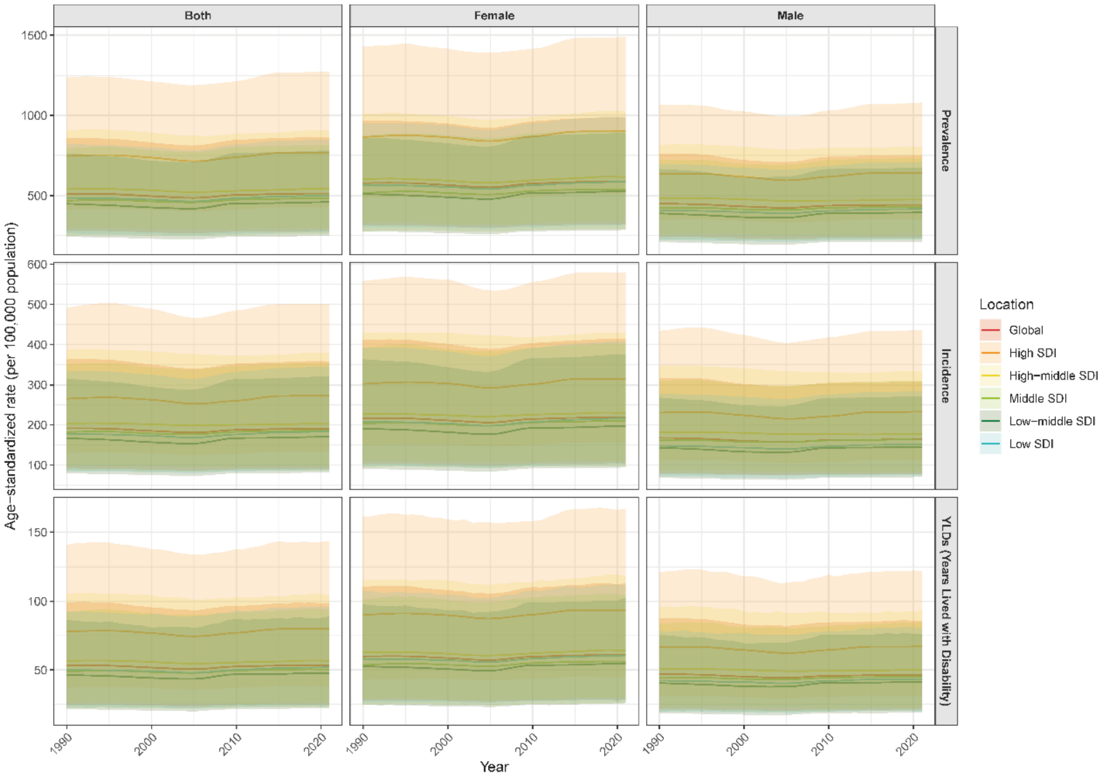

Supplementary Figure 5. Average annual percent changes of age standardized incidence, YLDs and prevalence of NP among children and adolescents from 1990 to 2021 at socio-demographic index levels by sex

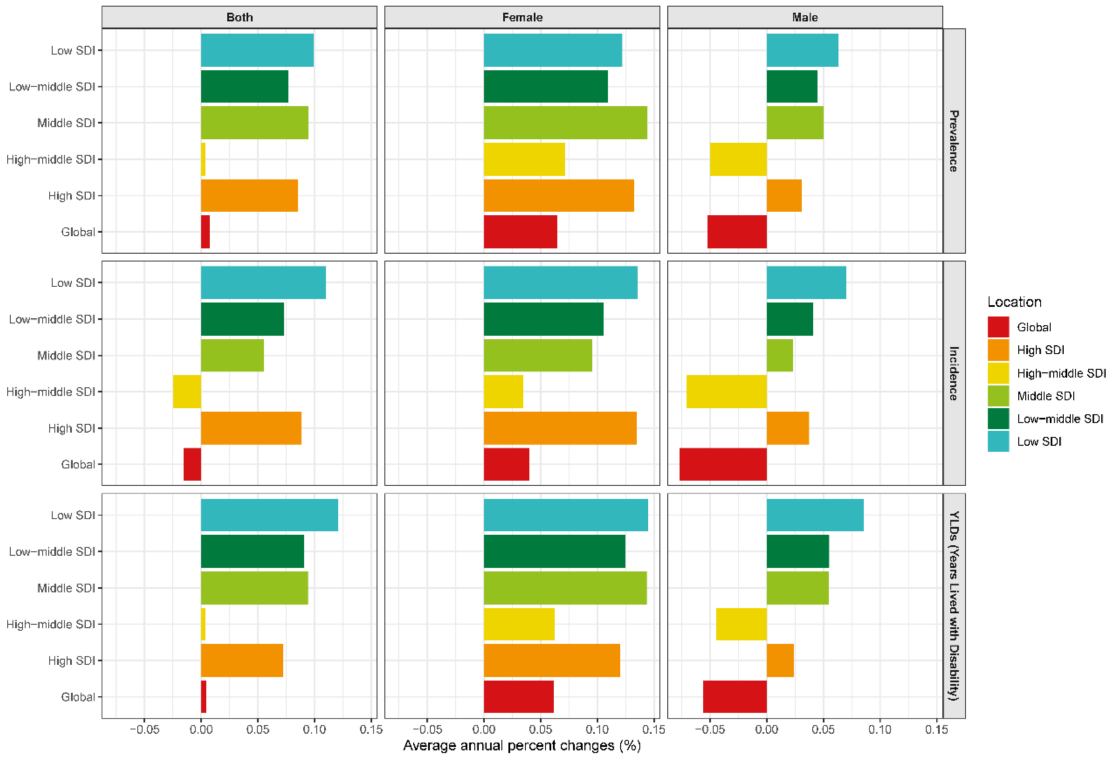

Supplementary Figure 6. Average annual percent changes of age standardized incidence, prevalence and YLDs of NP among children and adolescents from 1990 to 2021 by sex and age

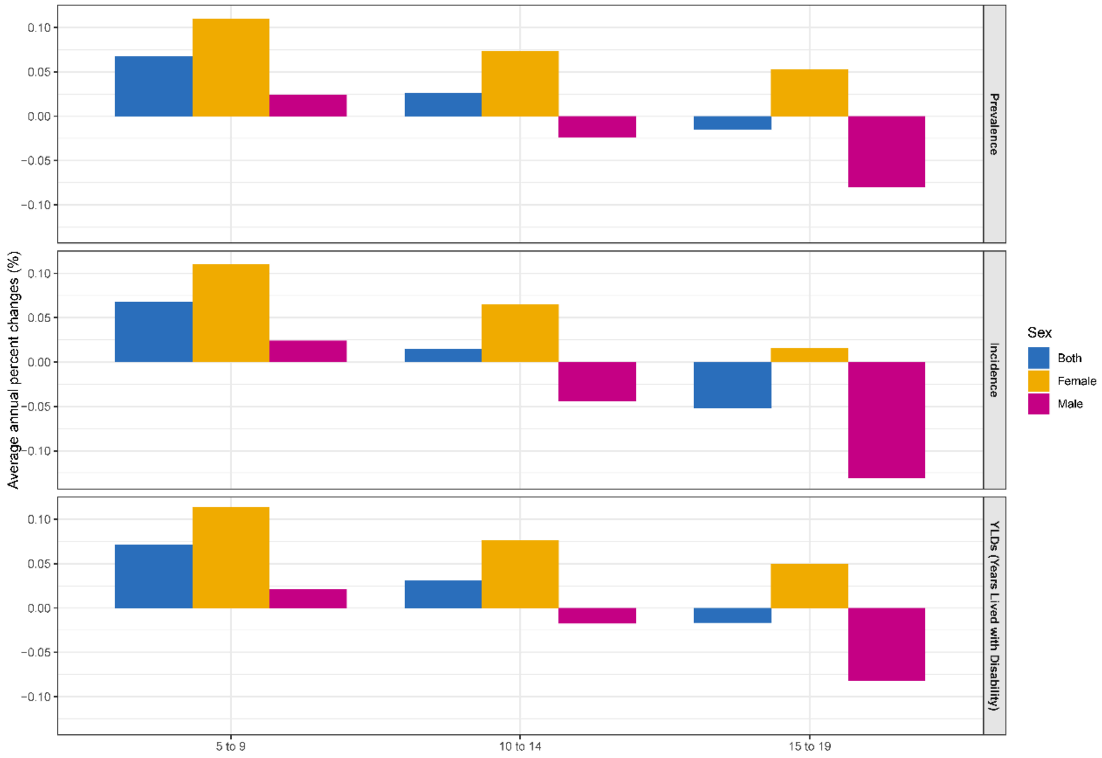

Supplementary Figure 7. Temporal trend of age standardized prevalence, incidence and YLDs of NP among children and adolescents and overall NP patients from 1990 to 2021 at global and socio-demographic index levels

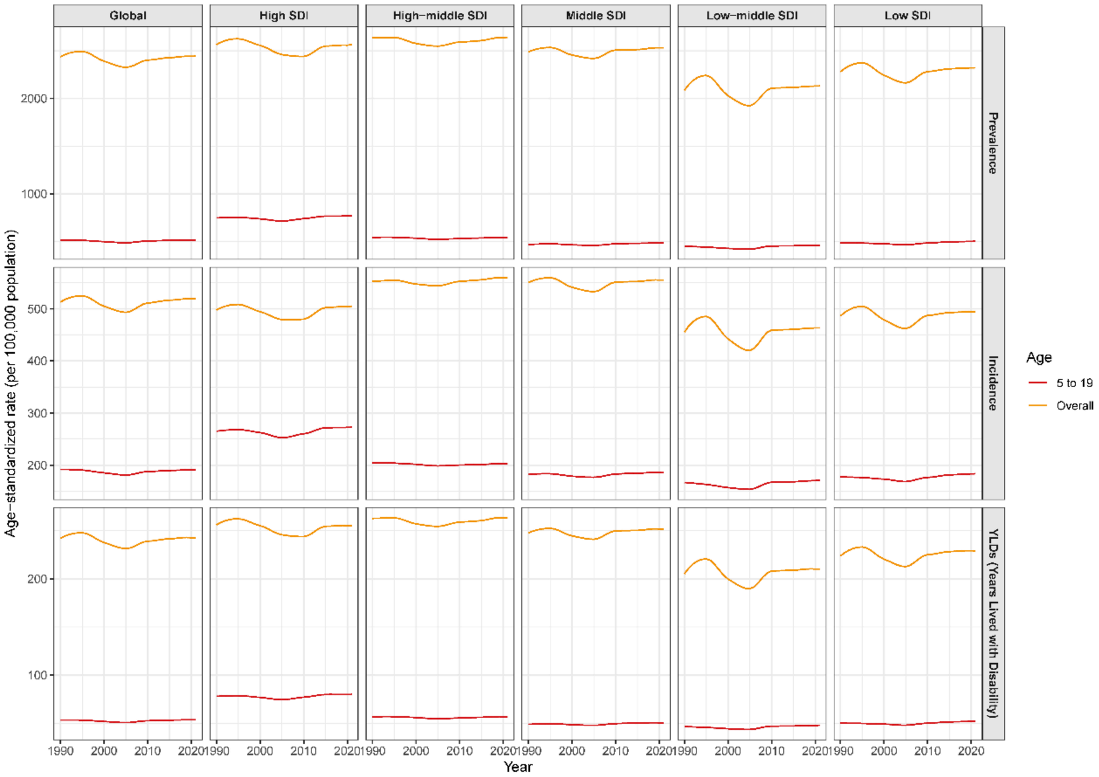

Supplementary Figure 8. Average annual percent changes of age standardized prevalence (A), incidence (B) and YLDs (C) of NP among children and adolescents and overall NP patients from 1990 to 2021 at global and socio-demographic index levels

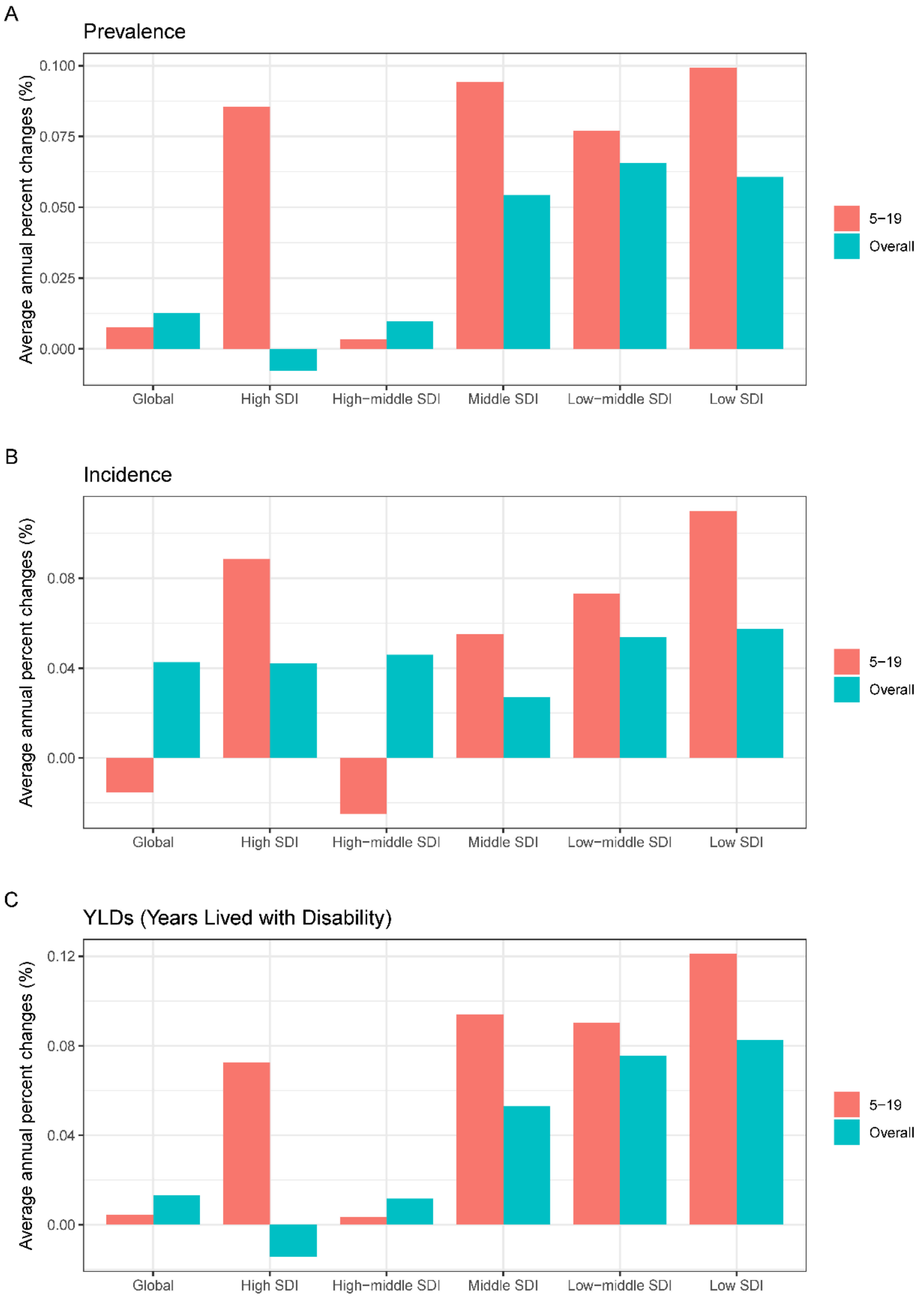

Supplementary Figure 9. Prevalence (A), incidence (B) and YLDs (C) rate of NP among children and adolescents from 204 countries according to the socio-demographic index in 2021

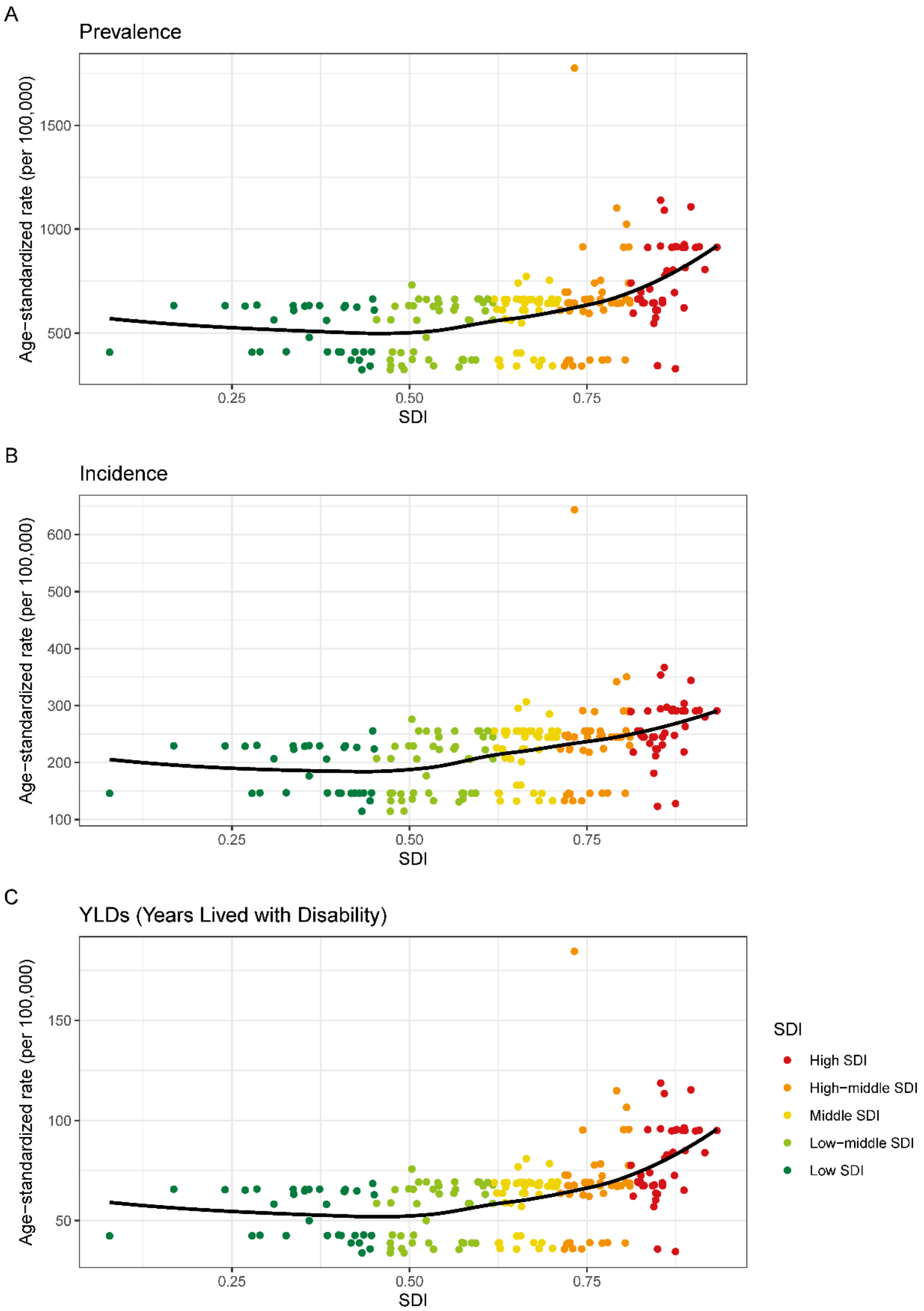

Supplementary Figure 10. Average annual percent changes of age standardized prevalence, incidence and YLDs of NP among children and adolescents from 1990 to 2021 at regions levels

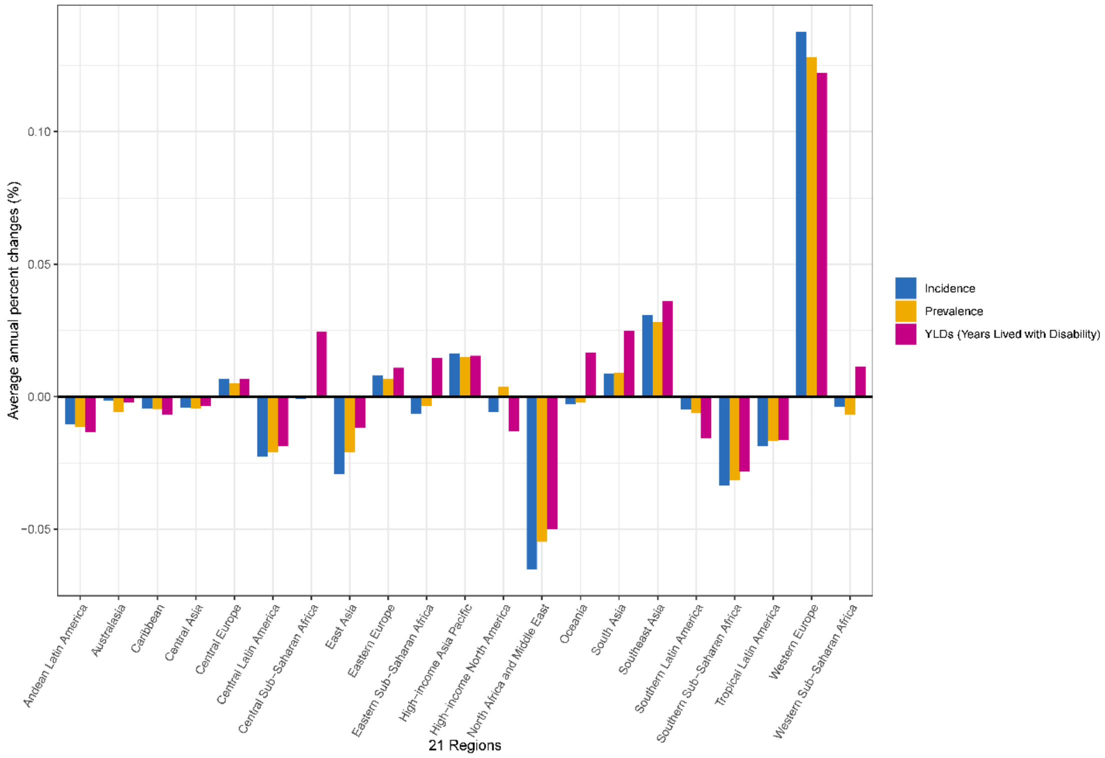

Supplementary Figure 11. Average annual percent changes of age-standardized incidence, YLDs and prevalence of NP among children and adolescents from 1990 to 2021 at regions levels by sex

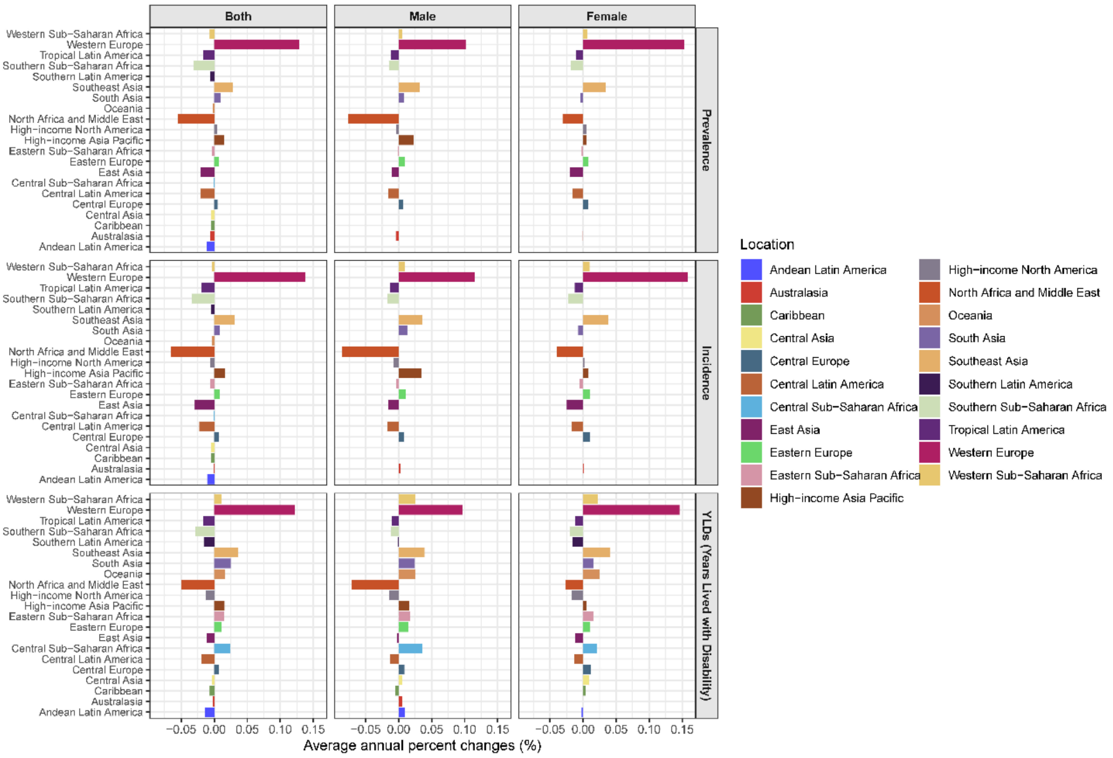

Supplement: Supplementary file 1 [file Data_Sheet_1.pdf]
